# Machine learning-based identification of inflammatory biomarkers for predicting pulmonary consolidation in children with Chlamydia pneumoniae infection

**DOI:** 10.3389/fped.2026.1779116

**Published:** 2026-05-04

**Authors:** Qianqian Dai, Zhiyuan Wang, Junlin Zhao, Yanan Wang, Menghua Li, Aliya Maimaitiniyazi, Xueli Wang, Jianjiang Cui, Zhenzhen Guo, Shengmeng Qu, Wen Zhao, Liang Ru

**Affiliations:** Department of Pediatrics, The First Affiliated Hospital of Xinjiang Medical University, Urumqi, Xinjiang, China

**Keywords:** children, Chlamydia pneumoniae, inflammatory biomarkers, machine learning, pulmonary consolidation, risk assessment

## Abstract

**Objective:**

This study aimed to identify core inflammatory biomarkers through machine learning approaches and develop an accessible online risk calculator to predict pulmonary consolidation in children with Chlamydia pneumoniae infection, addressing the current lack of effective early warning tools.

**Methods:**

This retrospective case-control study enrolled 42 children with C. pneumoniae infection (consolidation group: 26 cases; non-consolidation group: 16 cases) between January 2020 and December 2024. Five machine learning algorithms, including least absolute shrinkage and selection operator (LASSO) regression, support vector machine-recursive feature elimination (SVM-RFE), Random Forest, XGBoost, and LightGBM, were employed for feature selection, and core predictive factors were identified through consensus validation across these algorithms. K-means clustering analysis was performed on the key inflammatory markers, and an online risk assessment system based on HTML5 technology was developed.

**Results:**

The five machine learning algorithms consistently identified lactate dehydrogenase (LDH), C-reactive protein (CRP), and erythrocyte sedimentation rate (ESR) as core inflammatory markers for predicting pulmonary consolidation. All three indicators were significantly higher in the consolidation group compared with the non-consolidation group (*P* < 0.001). K-means clustering analysis stratified patients into a high-inflammation group (9 cases, 21.4%) and a low-inflammation group (33 cases, 78.6%), with a significant difference in consolidation rates between the two groups (100% vs. 51.5%, *P* = 0.008). The risk assessment system, constructed based on clustering results, demonstrated excellent predictive performance [area under the curve (AUC) = 0.993, 95% confidence interval (CI): 0.966–1.000], with a sensitivity of 88.9% and specificity of 93.9%. Bootstrap resampling validation (1,000 iterations) confirmed the robustness of the clustering solution (stability: 91.2%) and the risk assessment system (bootstrap AUC: 0.949, 95% CI: 0.881–0.995).

**Conclusion:**

LDH, CRP, and ESR are key indicators for predicting pulmonary consolidation in children with C. pneumoniae infection. The online risk assessment system developed based on these three routine laboratory parameters demonstrates good clinical usability and practicality, enabling early identification of high-risk patients to guide individualized treatment decisions.

## 1 Introduction

Chlamydia pneumoniae (C. pneumoniae) is one of the important atypical pathogen infections causing community-acquired pneumonia (CAP) in children. The literature reports that its overall proportion in pediatric CAP is typically around 1%–2%. However, in specific populations such as school-age children and adolescents, the detection rate can increase to 5%–20%. This variation depends on differences in study populations, geographic regions, and diagnostic methods (1, 2). Recent surveillance data suggest a resurgence of Chlamydia pneumoniae respiratory infections in certain regions. In Germany, analysis of multicenter nucleic acid testing data showed a significantly higher detection rate in 2024 than in 2019 (adjusted OR ≈ 3.03), with increases observed not only in children but also in adults aged 30–50 years, indicating renewed community circulation of this pathogen (3). Atypical pathogen infections in children often manifest diverse clinical courses. For instance, although Chlamydia trachomatis respiratory infection is relatively rare in children, a large-sample study demonstrated that its clinical manifestations can range from mild infection to severe pneumonia (4). In contrast, the more common C. pneumoniae infection typically follows a self-limiting course. Nevertheless, studies and case reports indicate that in severe or mixed infections in children, it can progress to severe pneumonia or even pulmonary consolidation, thereby increasing diagnostic and therapeutic complexity (5). Pulmonary consolidation, an important radiological marker of pneumonia severity, involves sustained inflammatory responses, alveolar exudation, and cascading amplification of tissue injury. However, there is currently a lack of effective early warning tools, such as biomarkers or clinical scoring systems, for predicting the risk of pulmonary consolidation in children with C. pneumoniae infection.

Traditional assessment of pneumonia severity mainly relies on clinical symptoms, signs, and single laboratory indicators; however, these methods have obvious limitations in the early identification of high-risk patients. Single biomarkers, such as procalcitonin (PCT), have limited discriminatory value in atypical pathogen infections (6). Moreover, predictive models integrating multiple inflammatory indicators have not been systematically validated in patients infected with C. pneumoniae. In recent years, machine learning technology has demonstrated significant potential in the field of pediatric pneumonia risk assessment and prediction. Zhang et al. developed a model for early diagnosis of severe pneumonia caused by Mycoplasma pneumoniae, based on multiple machine learning algorithms, and showed high predictive performance in the validation cohort. This suggests that integrating multiple clinical and laboratory indicators can improve predictive accuracy (7). Shen et al. used algorithms such as XGBoost to construct a machine learning model for predicting lobar pneumonia risk in children. The model showed excellent performance and identified key predictive factors through SHAP analysis, further demonstrating the application value of machine learning methods in pediatric CAP risk identification (8). However, despite these advances in M. pneumoniae research, studies focusing on risk factor identification and predictive model construction for pulmonary consolidation caused by C. pneumoniae infection remain scarce.

Inflammatory biomarkers play a key role in assessing pneumonia severity. Multiple studies have shown that indicators such as C-reactive protein (CRP), lactate dehydrogenase (LDH), and erythrocyte sedimentation rate (ESR) are closely related to severe pneumonia in children (9, 10). These markers not only reflect the magnitude of systemic inflammatory responses but also indirectly indicate the extent of lung tissue damage. Nonetheless, in the specific scenario of C. pneumoniae infection, which inflammatory markers have the highest predictive value for pulmonary consolidation remains unclear. In addition, how to identify core predictive factors through objective feature selection methods has not been fully elucidated. The multi-algorithm comparison and ensemble learning strategy in machine learning can evaluate variable importance from different perspectives, thereby improving the reliability of model construction. This strategy has been widely applied in recent studies. For example, Yang et al., when developing a predictive model for severe pneumonia in children, systematically applied and compared several machine learning algorithms, such as logistic regression, random forest, and XGBoost, to select key features and construct the optimal models (11). This methodological innovation provides new insights for feature selection in small-sample medical research.

Furthermore, unsupervised clustering analysis, a data-driven patient stratification method, can identify patient subgroups with similar inflammatory phenotypes without relying on preset labels, providing a basis for precision medicine and individualized treatment decisions. Whether clustering-based stratification using inflammatory markers can effectively identify high-risk populations for pulmonary consolidation and offer practical risk assessment tools for clinical decision-making is an urgent clinical question, as timely identification could significantly impact patient outcomes.

This study aims to systematically identify core inflammatory markers for pulmonary consolidation caused by Chlamydia pneumoniae infection in children by integrating multiple machine learning algorithms with unsupervised clustering analysis and to construct a risk assessment system based on routine laboratory indicators. The study screens key predictive factors through consensus validation of five machine learning algorithms. It then uses K-means clustering analysis to identify patient subgroups with different inflammatory phenotypes and develops an online risk calculator for rapid bedside risk stratification. Within this single-center retrospective cohort, our analysis provides preliminary evidence on inflammatory patterns associated with pulmonary consolidation in children with C. pneumoniae infection and presents an initial laboratory-based tool to facilitate bedside risk stratification; further validation is warranted in larger and external cohorts.

Table 1 summarizes representative studies employing machine learning approaches for pneumonia-related prediction in pediatric populations. While several studies have applied ML algorithms to predict severe M. pneumoniae pneumonia or lobar pneumonia, no prior study has specifically addressed the prediction of pulmonary consolidation in children with C. pneumoniae infection. The present study preliminarily explored the application of a multi-algorithm consensus approach to identify potential predictive biomarkers for pulmonary consolidation in children with C. pneumoniae infection.

**Table 1:** Comparison of machine learning-based prediction models for pneumonia outcomes in pediatric populations.

Study	Target Pathogen/Condition	Sample Size	Key Methods	Main Biomarkers/Features	Performance	Limitations
Xie et al. (7)	M. pneumoniae (SMPP vs. mild MPP)	*n* = 597	LASSO + 8 ML algorithms (LightGBM, RF, XGBoost, SVM, etc.); SHAP; prospective cohort validation; DCA and CIC	S100A8/A9, CT imaging score, RBP, P-LCR, Treg cell count (SCRPT simplified model)	LightGBM best: AUC = 0.92 (prospective validation); SCRPT simplified model AUC > 0.80	Single-center; required specialized biomarkers (S100A8/A9, Treg cells) not routinely available; no online clinical tool
Shen et al. (8)	Pediatric lobar pneumonia (CAP)	*n* = 278 (65 LP)	4 ML algorithms (LR, SVM, XGBoost, DT); Boruta feature selection; univariable LR; SHAP interpretation	Age, CRP, CD64 index, lymphocyte percentage, ALB (top 5 by SHAP)	XGBoost best: AUC = 0.880 (95% CI: 0.807–0.934) training; AUC = 0.746 (95% CI: 0.664–0.843) validation	Single-center; small sample; focused on CAP-related LP (not pathogen-specific); no multi-algorithm consensus; no external validation
Yang et al. (11)	Severe pneumonia (mixed pathogens, mixed ages)	*n* = 204 (7 centers)	6 ML algorithms (LR, RF, NB, XGBoost, SVM, DT); LASSO regression + clinical insight; multicenter design; SHAP	Age, sex, WBC, T-lymphocyte count, NLR, CRP, TNF-α, IL-4/IFN-γ ratio, IL-6/IL-10 ratio	XGBoost best: AUC = 0.901 (95% CI: 0.827–0.985) in test cohort; accuracy 0.803; sensitivity 0.844	Small sample (*n* = 204); not pediatric-specific (mixed-age cohort); required cytokine panels (TNF-α, interleukins) not routinely tested; oncology hospital-based
Ma et al. (13)	Pediatric SCAP to cSCAP progression	*n* = 211	LASSO + LR variable screening; 7 ML algorithms (LR, DT, RF, XGBoost, NB, KNN, SVM); SHAP interpretation	RDW-CV, PCT, BUN, LDH (4 predictors selected)	XGBoost best: AUC = 0.98 (95% CI: 0.93–1.00); accuracy 0.89; sensitivity 0.98; specificity 0.75	Single-center; PICU-only cohort with potential selection bias; no external validation; no online clinical tool
Ling et al. (14)	M. pneumoniae identification in segmental/lobar pneumonia	*n* = 630	4 ML algorithms (LR, DT, RF, KNN); feature selection from 11 clinical variables; 7 variables retained for modeling	Selected from: age, WBC, CRP, NLR, PLR, LDH, pneumonia volume %, pulmonary complications, etc. (7 of 11 initial features)	RF best: AUC = 0.752; accuracy 69.6%; sensitivity 57.1%	Single-center; retrospective (Dec 2016–Dec 2021); moderate predictive performance; no SHAP analysis; no online clinical tool
**This study**	***C. pneumoniae* pulmonary consolidation**	***n* = 42**	**5 ML algorithms (LR, RF, SVM, XGBoost, LightGBM); multi-algorithm consensus; K-means clustering; SHAP; bootstrap validation; online risk calculator**	**LDH, CRP, ESR, fever duration, age (identified by multi-algorithm consensus)**	**Multi-algorithm consensus validation; bootstrap internal validation; integrated unsupervised clustering for risk stratification**	**Single-center; retrospective; preliminary sample size; external validation pending; specific to C. pneumoniae infection**

ML, machine learning; LR, logistic regression; RF, random forest; SVM, support vector machine; XGBoost, extreme gradient boosting; LightGBM, light gradient boosting machine; DT, decision tree; NB, Naïve Bayes; KNN, k-nearest neighbor; LASSO, least absolute shrinkage and selection operator; SHAP, shapley additive explanations; SMPP, severe mycoplasma pneumoniae pneumonia; MPP, mycoplasma pneumoniae pneumonia; CAP, community-acquired pneumonia; SCAP, severe community-acquired pneumonia; cSCAP, critical severe community-acquired pneumonia; LP, lobar pneumonia; LDH, lactate dehydrogenase; CRP, C-reactive protein; ESR, erythrocyte sedimentation rate; PCT, procalcitonin; WBC, white blood cell; NLR, neutrophil-to-lymphocyte ratio; PLR, platelet-to-lymphocyte ratio; RDW-CV, red cell distribution width–coefficient of variation; BUN, blood urea nitrogen; RBP, retinol-binding protein; P-LCR, platelet large cell ratio; ALB, albumin; AUC, area under the receiver operating characteristic curve; DCA, decision curve analysis; CIC, clinical impact curve.

Bold text in the last row indicates the present study. Reference numbers correspond to the manuscript reference list. All data were verified from the original full-text publications.

In summary, the main contributions of this study are as follows: (1) We applied and compared five machine learning–based feature selection approaches to identify inflammatory biomarkers that were consistently selected for predicting pulmonary consolidation in children with C. pneumoniae infection. (2) We further used unsupervised clustering based on the selected routine laboratory markers to characterize inflammatory phenotypes and explored their association with pulmonary consolidation within this cohort. (3) Based on the above findings, we developed a preliminary risk assessment approach using routinely available laboratory indicators and implemented it as an online calculator to facilitate rapid risk stratification, while noting that further validation is warranted.

The remainder of this paper is organized as follows. Section 2 describes the study design, participants, variables, and the analytical workflow, including feature selection, clustering, and model development. Section 3 reports the main results of group comparisons and model performance. Section 4 discusses the findings in the context of existing evidence and outlines limitations and future directions. Finally, Section 5 concludes the study.

## 2 Materials and methods

### 2.1 Study design and participants

**Figure 1:**
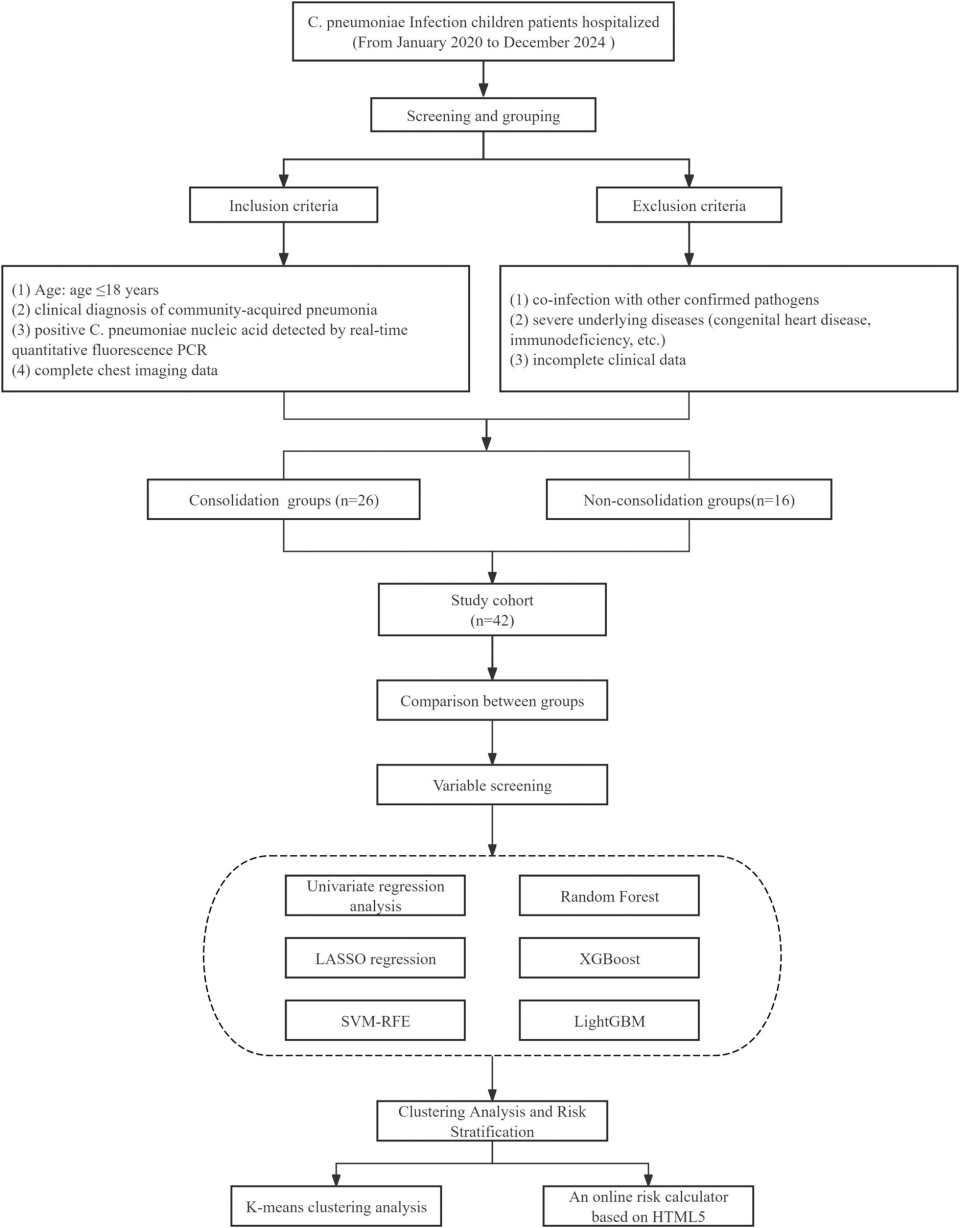
Flowchart of case selection and modeling process.

This retrospective case-control study enrolled 42 children with C. pneumoniae infection (the consolidation group: 26 cases; the non-consolidation group: 16 cases) admitted to the Department of Pediatrics of our hospital between January 2020 and December 2024 (Figure 1). Inclusion and exclusion criteria were based on the “Guidelines for the management of community-acquired pneumonia in children (2024 revision)” (12).Inclusion criteria were as follows: age ≤18 years; clinical diagnosis of community-acquired pneumonia; positive C. pneumoniae nucleic acid detected by real-time quantitative fluorescence PCR; complete chest imaging data. Exclusion criteria were as follows: co-infection with other confirmed pathogens; severe underlying diseases (congenital heart disease, immunodeficiency); incomplete clinical data. Pulmonary consolidation was defined as homogeneously increased lung tissue density, prominent air bronchograms, and lesions involving one or more pulmonary segments. This was confirmed by consensus between two radiologists with more than 5 years of experience independently reviewing the images.

### 2.2 Data collection

Demographic characteristics such as gender and age were collected through the electronic medical record system. Clinical manifestations including disease duration, cough characteristics, fever, hypoxemia, and allergy history were also recorded. Data on pathogen detection, laboratory tests, imaging features, and complications were gathered accordingly. Laboratory tests included complete blood count (CBC), CRP, interleukin-6 (IL-6), procalcitonin (PCT), ESR, LDH, D-dimer, and immunoglobulin E (IgE). Specimen collection was performed from pharyngeal swabs, sputum, or bronchoalveolar lavage fluid, and C. pneumoniae nucleic acid was detected by real-time quantitative fluorescence PCR. Both DNA copy number and DNA concentration were calculated according to the standard curve.

### 2.3 Statistical analysis

IBM SPSS Statistics version 29.0 (IBM Corp., Armonk, NY, USA) was used for basic statistical analysis. Continuous variables were expressed as median (interquartile range, Q_1_–Q_3_), and comparisons between groups were performed using the Mann–Whitney U test. Categorical variables were expressed as numbers (percentages) [*n* (%)], and comparisons between groups were performed using the chi-square (*χ*²) test or Fisher's exact test. Univariate logistic regression analysis included variables with statistically significant baseline differences (*P* < 0.05) and disease-related clinical indicators. This analysis was used to calculate odds ratios (OR) and 95% confidence intervals (CI). Two-tailed hypothesis tests were performed, with *P* < 0.05 considered statistically significant. Given the relatively small sample size (*n* = 42), several strategies were employed to mitigate overfitting and enhance the reliability of findings: (1) Non-parametric statistical methods (Mann–Whitney U test, Fisher's exact test) were used for group comparisons, which do not assume normal distribution and are more appropriate for small samples. (2) Robust standardization using median and interquartile range (rather than mean and standard deviation) was applied for Z-score calculation, which is less sensitive to outliers. (3) Regularization techniques were incorporated in machine learning algorithms (L1 regularization in LASSO, L1/L2 regularization in LightGBM) to prevent overfitting. (4) The consensus feature selection strategy, requiring agreement across five independent algorithms, reduces the likelihood of identifying spurious associations. (5) Stratified cross-validation was used to maintain class balance in each fold.

### 2.4 Machine learning feature selection

Machine learning analysis was performed using R software (v4.5.2) with a random seed set to 12,345 for reproducibility. After removing treatment-related variables from the initial set of 52 candidate variables, 42 variables were included in the analysis (Supplementary Table S1). Prior to machine learning feature selection, a pre-screening step was performed to exclude variables that could introduce multicollinearity or circularity in prediction. Three absolute cell count variables (neutrophil count, lymphocyte count, and eosinophil count) were removed because their corresponding percentages were already included, and retaining both would introduce strong collinearity. Twelve imaging-related variables [involvement of individual lung lobes (right upper, right middle, right lower, left upper, left lower), number of lobes involved, bilateral lung involvement, ground-glass opacity, nodule, patchy shadow, air bronchogram, and pleural effusion] were excluded because these features represent concurrent radiological findings rather than antecedent predictors; including them would create circularity, as pulmonary consolidation is itself defined by imaging criteria. After this pre-screening, 27 variables were retained for input into SVM-RFE, Random Forest, XGBoost, and LightGBM. LASSO regression was applied to all 42 candidate variables, as L1 regularization inherently handles collinearity by shrinking redundant coefficients to zero.

For each algorithm, cross-validation served a dual purpose: (1) evaluating the generalization performance of the feature selection process by assessing prediction accuracy on held-out folds, and (2) optimizing algorithm-specific hyperparameters through grid search or regularization path evaluation within the cross-validation framework. Specifically, the optimal hyperparameter configuration was selected based on the best average performance metric across validation folds, thereby reducing the risk of overfitting to the training data. The detailed hyperparameter search spaces and optimal values for each algorithm are described below and summarized in Supplementary Table S2. Five algorithms were used for feature selection:

1. LASSO regression: Features were screened through L1 regularization and 10-fold cross-validation, and core variables were determined using the “lambda.1se” criterion, with the glmnet package (v4.1–8); The regularization parameter lambda was evaluated across a sequence of values automatically generated by glmnet along the full regularization path. The optimal lambda was selected using two criteria: lambda.min (minimizing cross-validated deviance, yielding 7 variables) and lambda.1se (the most regularized model within one standard error of the minimum deviance, yielding 3 core variables). The lambda.1se criterion was adopted for the final model to prioritize parsimony and generalizability.
2. SVM-RFE: Linear kernel SVM recursive feature elimination was applied, with 10-fold cross-validation to evaluate performance, using the e1071 package (v1.7–13); The cost parameter (C) was optimized via grid search over the candidate set {0.1, 1, 10} using the tune() function. An outer 10-fold cross-validation was used to assess generalization performance, while an inner 10-fold cross-validation within each fold was used to evaluate feature subsets during recursive elimination. Features were ranked based on their average importance rank across all outer cross-validation folds.
3. Random Forest: Feature importance was evaluated based on Gini impurity decrease (threshold 2.0), with 10-fold cross-validation to optimize hyperparameters, using the randomForest package; The number of candidate splitting variables (mtry) was searched over multiple values, with an optimal mtry of 5 selected. The number of trees (ntree) was set to 500, with out-of-bag (OOB) error stabilizing at approximately 200 trees. Features with a mean decrease in Gini impurity ≥ 2.0 were classified as high-importance predictors.
4. XGBoost: Feature Gain values were evaluated, with 5-fold cross-validation to optimize hyperparameters, using the xgboost package via the caret framework; Features with Gain > 0.01 were retained as informative predictors, yielding 9 variables for downstream analysis. Hyperparameters were optimized through grid search over the following candidate sets: number of boosting rounds (nrounds): {100, 200}; maximum tree depth (max_depth): {3, 5, 7}; learning rate (eta): {0.01, 0.1, 0.3}. The remaining parameters were held constant (gamma = 0, colsample_bytree = 1, min_child_weight = 1, subsample = 1). The optimal combination was selected based on the highest average AUC across the 5 validation folds.
5. LightGBM: Hyperparameters were optimized for small samples using 5-fold stratified cross-validation to determine optimal parameters. Features were screened based on Gain > 0.01 or ranking in the top 15, using the lightgbm package.

To enhance model interpretability and provide qualitative evidence for the identified features, SHapley Additive exPlanations (SHAP) analysis was performed on the XGBoost model using the shap package (v0.44) in Python (v3.x) with the same random seed (12,345) and optimal hyperparameters. A TreeExplainer was applied to compute exact SHAP values for all 42 patients. Four visualizations were generated: a global mean |SHAP| bar chart, a beeswarm summary plot, SHAP dependence plots for LDH, CRP, and ESR with Cluster-2 reference medians overlaid, and a SHAP heatmap for the top 15 features across all patients.

### 2.5 Clustering analysis and risk stratification

K-means clustering analysis was performed on the identified core inflammatory markers (LDH, CRP, ESR) with data standardization. The optimal number of clusters was comprehensively determined through the elbow method, the silhouette coefficient method, the gap statistic, the NbClust voting method, and the Calinski-Harabasz index. Clustering quality was evaluated using an appropriate metric, and Fisher's exact test was used to analyze the association between cluster grouping and pulmonary consolidation.

Based on the clustering results, Z-score standardization was performed using the low-inflammation group as the reference, meaning that Z-scores were calculated relative to the mean and standard deviation of this group. The comprehensive risk score was defined as the sum of Z-scores for the three indicators. The optimal threshold of the comprehensive risk score (3.75) was determined through ROC curve analysis and the Youden index. An online risk calculator based on HTML5 was developed to facilitate risk assessment. The Cluster, factoextra, and NbClust packages were used to complete the clustering analysis.

To assess the stability and robustness of the clustering solution and risk assessment system, bootstrap resampling validation was performed. A total of 1,000 bootstrap samples were generated by sampling with replacement from the original cohort (*n* = 42). For each bootstrap sample, K-means clustering (*k* = 2) was repeated with identical hyperparameters, and the proportion of patients consistently assigned to the same cluster across iterations was calculated as a measure of cluster stability. The 95% confidence intervals (CIs) for the AUC, sensitivity, and specificity of the composite risk score were estimated from the bootstrap distribution using the bias-corrected and accelerated (BCa) method.

Sensitivity analyses were performed to further evaluate result robustness. First, leave-one-out cross-validation (LOOCV) was applied: each patient was sequentially excluded (*n* = 42 iterations) and K-means clustering was re-run on the remaining 41 patients, with cluster composition compared to the original solution. Second, random seed sensitivity was assessed by repeating clustering across 100 different random seeds (1–100); a run was classified as yielding the same solution if ≥90% of patients were assigned to the same cluster as in the original analysis. Third, threshold sensitivity was examined by evaluating sensitivity, specificity, positive predictive value (PPV), and negative predictive value (NPV) of the composite risk score across a range of thresholds (Youden-optimal threshold ± 1.0, in increments of 0.5).

## 3 Results

### 3.1 Comparison of clinical data between groups sample characteristics

A total of 42 children with C. pneumoniae infection were enrolled, including 26 cases in the consolidation group and 16 cases in the non-consolidation group. There were no significant differences between the two groups in gender distribution, age, clinical symptoms, allergy history, and pathogen load. The duration of illness before admission in the consolidation group was significantly longer than that in the non-consolidation group [10.00 (6.00, 14.00) d vs. 6.00 (5.25, 9.25) d, *P* = 0.045]. Laboratory tests showed that the consolidation group had significantly higher white blood cell counts [10.76 (9.93, 13.98) vs. 8.64 (7.60, 9.89) × 10⁹/L, *P* = 0.001]; higher neutrophil percentage [79.15 (70.10, 84.16)% vs. 60.44 (58.12, 72.58)%, *P* < 0.001); significantly lower lymphocyte percentage [16.63 (13.55, 20.40)% vs. 24.84 (16.00, 29.26)%, *P* = 0.029]; significantly higher neutrophil-to-lymphocyte ratio (NLR) [4.69 (3.38, 6.07) vs. 2.43 (1.86, 4.58), *P* = 0.008]; and significantly higher platelet counts [343.50 (321.25, 394.50) vs. 296.50 (246.00, 341.75) × 10⁹/L, *P* = 0.048]. Regarding inflammatory markers, the consolidation group had significantly higher C-reactive protein [26.80 (22.71, 51.38) vs. 17.00 (15.00, 23.25) mg/L, *P* < 0.001], erythrocyte sedimentation rate [30.42 (23.05, 39.82) vs. 19.29 (13.58, 22.13) mm/h, *P* < 0.001], interleukin-6 [15.37 (13.01, 24.46) vs. 12.41 (11.39, 15.33) pg/mL, *P* = 0.045], and lactate dehydrogenase [319.66 (265.11, 372.41) vs. 218.62 (172.96, 274.34) U/L, *P* < 0.001]. Procalcitonin, D-dimer, and immunoglobulin E levels showed no significant differences between groups.

Imaging examinations showed no significant differences between the two groups in the distribution of affected lung lobes, the number of involved lobes, and imaging manifestations. Regarding treatment, antibiotic use was similar between the two groups, but the consolidation group had a significantly longer duration of corticosteroid use compared with the non-consolidation group [4.00 d (range, 0.00–5.00 d) vs. 0.00 d (range, 0.00–4.00 d), *P* = 0.022]. There were no significant differences in the incidence of extrapulmonary symptoms, pleural effusion, and complications, such as allergic rhinitis, between the two groups. Detailed data for these results are shown in Supplementary Table S3.

### 3.2 Univariate logistic regression analysis of significant risk factors for pulmonary consolidation

Univariate logistic regression showed that prolonged disease duration at admission (OR = 1.158, *P* < 0.001), corticosteroid use (OR = 2.506, *P* = 0.003), allergic rhinitis (OR = 1.945, *P* = 0.036), elevated white blood cell-related indicators—specifically WBC count, neutrophil count and percentage, and NLR (all *P* < 0.05)—decreased lymphocyte percentage (OR = 0.943, *P* = 0.002), elevated inflammatory markers—CRP, IL-6, ESR, and LDH (all *P* < 0.001)—and an increased number of involved lobes (OR = 1.759, *P* < 0.001) were associated with pulmonary consolidation. In contrast, air bronchogram was a protective factor (OR = 0.412, *P* = 0.012). Detailed data are shown in Supplementary Table S4.

### 3.3 Risk factor variable screening using five machine learning methods

To identify the most informative predictive variables for pulmonary consolidation, we applied five machine learning algorithms for feature selection. Each algorithm evaluates variable importance from a different perspective: LASSO performs L1 regularization-based selection, SVM-RFE uses recursive elimination, and tree-based models (Random Forest, XGBoost, LightGBM) assess features based on split gain. The following subsections present the results of each algorithm, and the consensus features identified across all methods are summarized at the end of this section.

**Figure 2:**
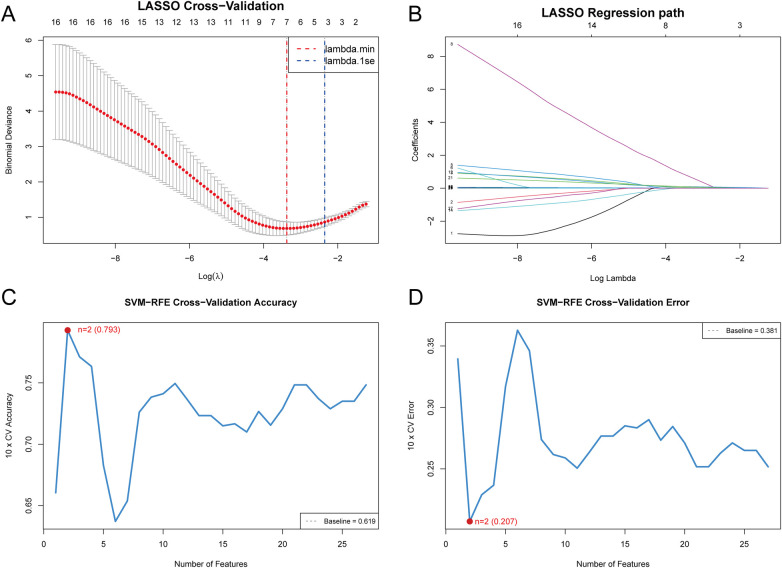
Identification of optimal diagnostic variables through LASSO regression and support vector machine recursive feature elimination. **(A)** Cross-validation curve of LASSO regression displaying the partial likelihood deviance as a function of log(λ), with vertical dashed lines indicating lambda.min and lambda.1se for optimal model selection. **(B)** LASSO coefficient regularization paths illustrating the shrinkage of variable coefficients toward zero with increasing regularization parameter λ, enabling systematic feature selection. **(C)** SVM-RFE classification accuracy plotted against the number of retained features, demonstrating peak predictive performance at *n* = 2 features. **(D)** SVM-RFE cross-validation error rates across varying feature subset sizes, confirming the optimal feature number with minimum error at *n* = 2.

**Figure 3:**
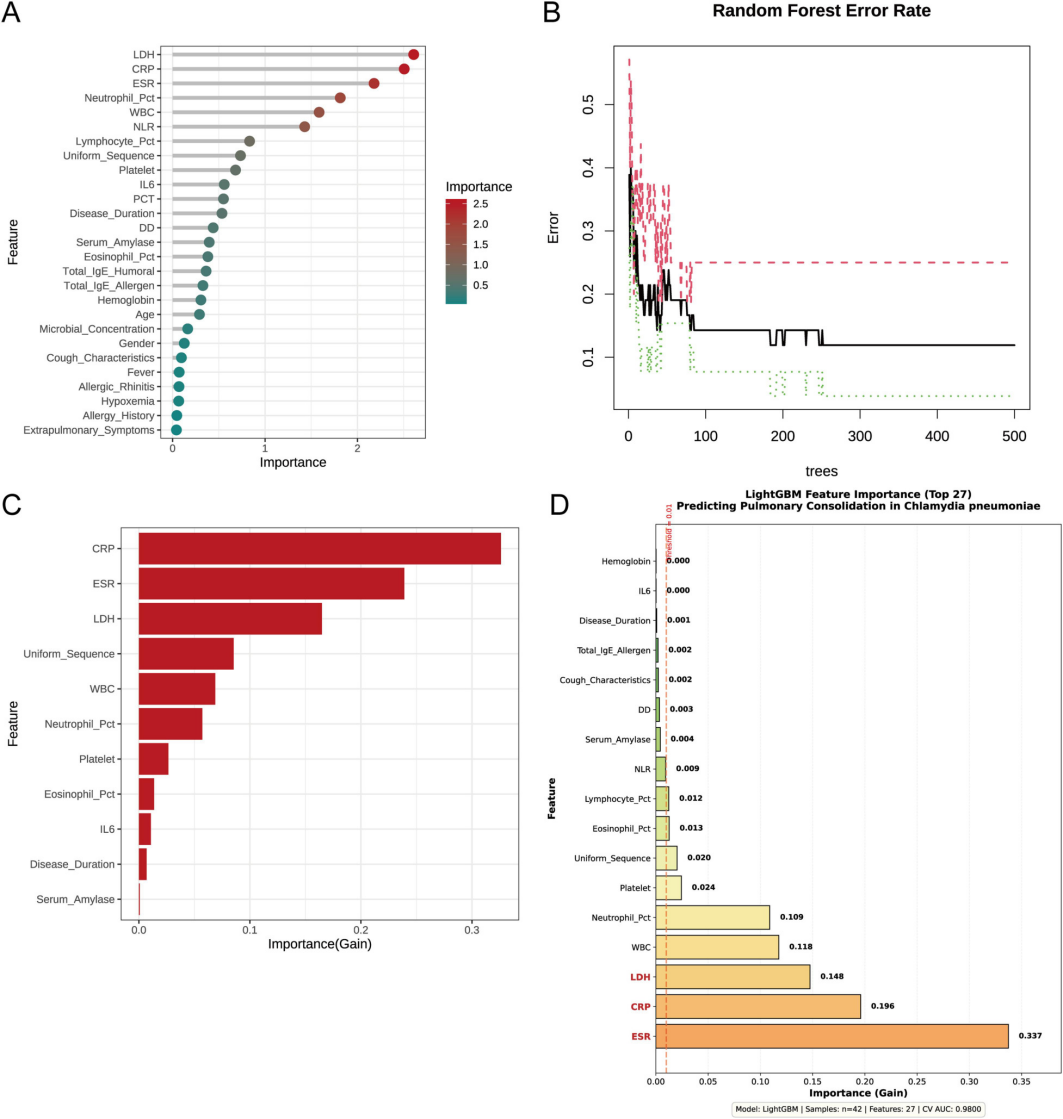
Identification of key diagnostic variables through random forest, XGBoost, and LightGBM algorithms. **(A)** Random Forest feature importance ranking based on mean decrease in Gini impurity, with the most contributory variables highlighted in the top positions. **(B)** Out-of-bag (OOB) error rate stabilization curve across increasing numbers of decision trees, demonstrating model convergence and robustness. **(C)** XGBoost feature importance plot displaying the top 9 variables with Gain > 0.01, ranked by the Gain metric reflecting each feature's contribution to model prediction. CRP, ESR, and LDH emerged as the dominant predictors, collectively accounting for 73.0% of the total Gain. **(D)** LightGBM feature importance ranking of 27 evaluated features. The top 15 were selected by a composite criterion (Gain > 0.01 or top-15 rank), stratified into three tiers: high importance (Gain ≥ 0.1, *n* = 5: ESR, CRP, LDH, WBC, Neutrophil_Pct), medium (0.01 ≤ Gain < 0.1, *n* = 4), and low (Gain < 0.01, *n* = 18). The top three contributors—ESR (0.337), CRP (0.196), and LDH (0.148)—accounted for 68.1% of cumulative Gain.

1. LASSO Regression Feature Selection: Among 42 candidate variables, 16 variables entered the regularization path through LASSO regression analysis. Complete variable selection results and model performance comparisons are shown in Supplementary Tables S5, S6. Ten-fold cross-validation showed that, under the “lambda.min” criterion, seven variables were selected, including disease duration at admission (coefficient = 0.041), neutrophil percentage (coefficient = 0.097), ESR (coefficient = 0.089), and LDH (coefficient = 0.013). Using the more stringent *lambda.1se* criterion, three core predictive variables were finally retained: neutrophil percentage (coefficient = 0.061), ESR (coefficient = 0.027), and LDH (coefficient = 0.0073). The cross-validation curve is shown in Figure 2A and the coefficient path in Figure 2B.
2. SVM-RFE: Cross-validation showed that model accuracy first increased and then stabilized as the number of features increased, reaching a peak of 0.793 when the number of features was 2 (Figure 2C). The cross-validation error curve (Figure 2D) exhibited a similar but inverse pattern, with the error rate decreasing to its lowest value of 0.207 when the number of features was 2. Ultimately, two core predictive variables were identified: neutrophil percentage and ESR (average rank = 3.0). The complete ranking of all 27 features is shown in Supplementary Table S7. These two variables were also among the three variables selected by LASSO regression, demonstrating the consistency between the two algorithms.
3. Random Forest: Feature importance was assessed for 27 variables. The data were converted to binary classification and standardized. 10-fold cross-validation was used to determine the optimal hyperparameters, with mtry = 5 and ntree = 500. Based on the average Gini impurity decrease, the importance ranking of 27 features is shown in Figure 3A (detailed in Supplementary Table S8). High-importance features (threshold ≥ 2.0) included 3 key predictive factors: LDH (2.608), CRP (2.506), and ESR (2.179). Medium-importance features (1.0–2.0) included neutrophil percentage (1.814), white blood cell count (1.585), NLR (1.429), and three other inflammatory indicators. The out-of-bag (OOB) error rate stabilized at approximately 200 trees (Figure 3B).
4. XGBoost: The optimal hyperparameters were nrounds = 100, max_depth = 3, and eta = 0.1. Using these optimal hyperparameters, the feature importance ranking showed that the top three features had a cumulative contribution of 73.0% in terms of Gain: CRP (Gain = 0.326), ESR (Gain = 0.239), and LDH (Gain = 0.165). A total of 9 features with Gain > 0.01 were retained for further analysis (Figure 3C, Supplementary Table S9). The complete set of hyperparameter settings is shown in Supplementary Table S10.
5. LightGBM: Feature importance was assessed for 27 variables. Hyperparameters were optimized for small-sample data, with num_leaves ranging from 5 to 15, learning_rate between 0.01 and 0.03, min_data_in_leaf from 3 to 8, and L1 and L2 regularization parameters both set to 0.5. A 5-fold stratified cross-validation grid search was used to determine the optimal parameters, which were as follows: num_leaves = 5, learning_rate = 0.01, and min_data_in_leaf = 3. Based on Gain values, the complete importance ranking of the 27 features is shown in Supplementary Table S11. Five core predictive factors with high importance (Gain ≥ 0.1) were identified, with the top three features contributing cumulatively 68.1%: ESR (Gain = 0.337), CRP (Gain = 0.196), and LDH (Gain = 0.148). A total of 15 features with Gain > 0.01 or ranking within the top 15 were selected for further analysis (Figure 3D).

**Figure 4:**
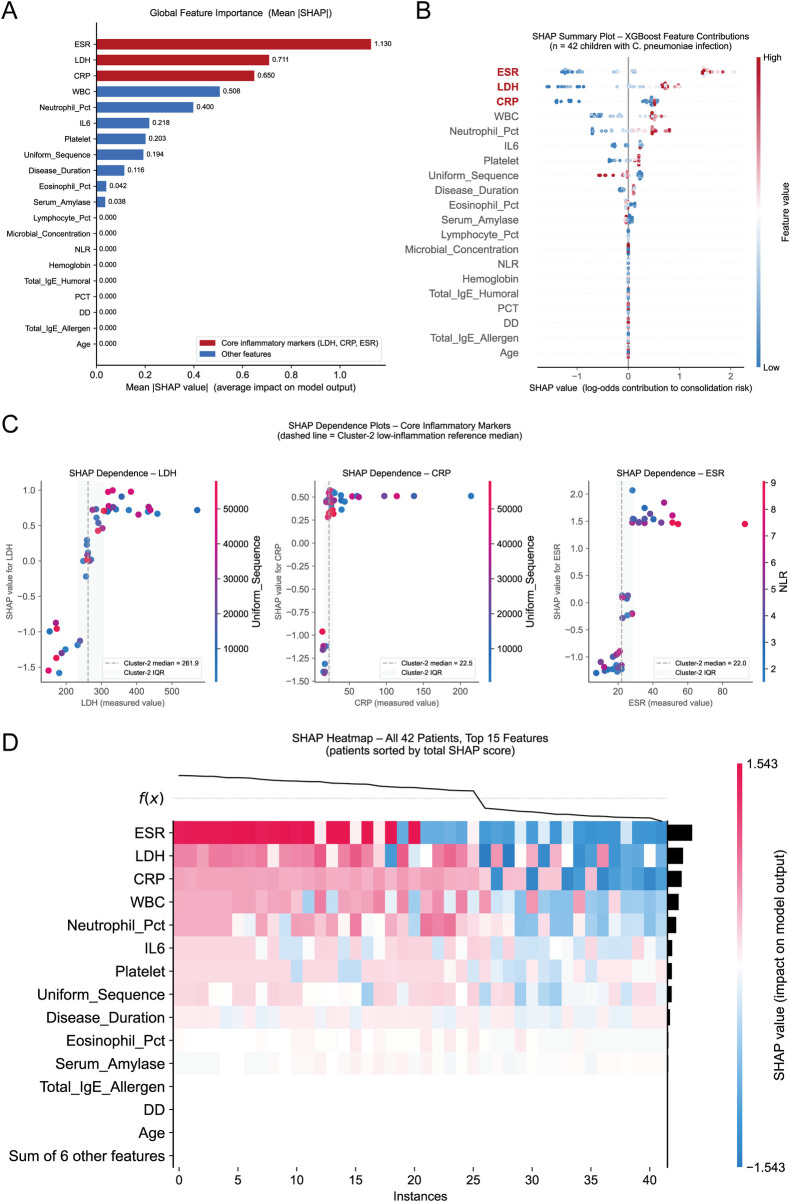
SHAP interpretability analysis of the XGBoost model for predicting pulmonary consolidation in children with C. pneumoniae infection. **(A)** Global feature importance ranked by mean |SHAP| values. ESR (1.130), LDH (0.711), and CRP (0.650) were the three dominant predictors, while seven features including Age, PCT, and D-dimer showed negligible contributions (Mean |SHAP| = 0). Core inflammatory markers are highlighted in red. **(B)** SHAP summary beeswarm plot showing the direction and magnitude of each feature's contribution across all 42 patients. Elevated values (red) of ESR, LDH, and CRP consistently increased consolidation risk (positive SHAP), while low values (blue) reduced risk. Lymphocyte_Pct showed an inverse pattern. **(C)** SHAP dependence plots for the three core inflammatory markers, with Cluster-2 low-inflammation reference medians overlaid as dashed lines. LDH showed a risk transition near 261.9 U/L, CRP near 22.5 mg/L, and ESR near 22.0 mm/h, corresponding to the low-inflammation cluster medians. **(D)** SHAP heatmap across all 42 patients (sorted by total SHAP score) for the top 15 features, illustrating individual-level contribution patterns and the clear separation between high- and low-risk patients.

SHAP Interpretability Analysis: SHAP analysis of the XGBoost model confirmed and extended the feature importance findings. Globally, ESR (Mean |SHAP| = 1.130), LDH (0.711), and CRP (0.650) were the three dominant predictors, collectively accounting for the majority of model output variation, while seven features including Age, PCT, and D-dimer contributed negligibly (Mean |SHAP| = 0) (Figure 4A). The beeswarm plot revealed consistent positive directionality for ESR, LDH, and CRP—elevated values increased consolidation risk—whereas Lymphocyte_Pct exhibited an inverse pattern (Figure 4B). SHAP dependence plots identified clinically interpretable risk thresholds: LDH near 261.9 U/L, CRP near 22.5 mg/L, and ESR near 22.0 mm/h (Figure 4C), each closely corresponding to the Cluster-2 low-inflammation group medians, thereby providing independent model-based validation of the clustering-derived risk boundaries. The patient-level SHAP heatmap further demonstrated clear separation between high- and low-risk individuals across the top 15 features (Figure 4D).

### 3.4 Clustering analysis of risk factors for pulmonary consolidation

**Figure 5:**
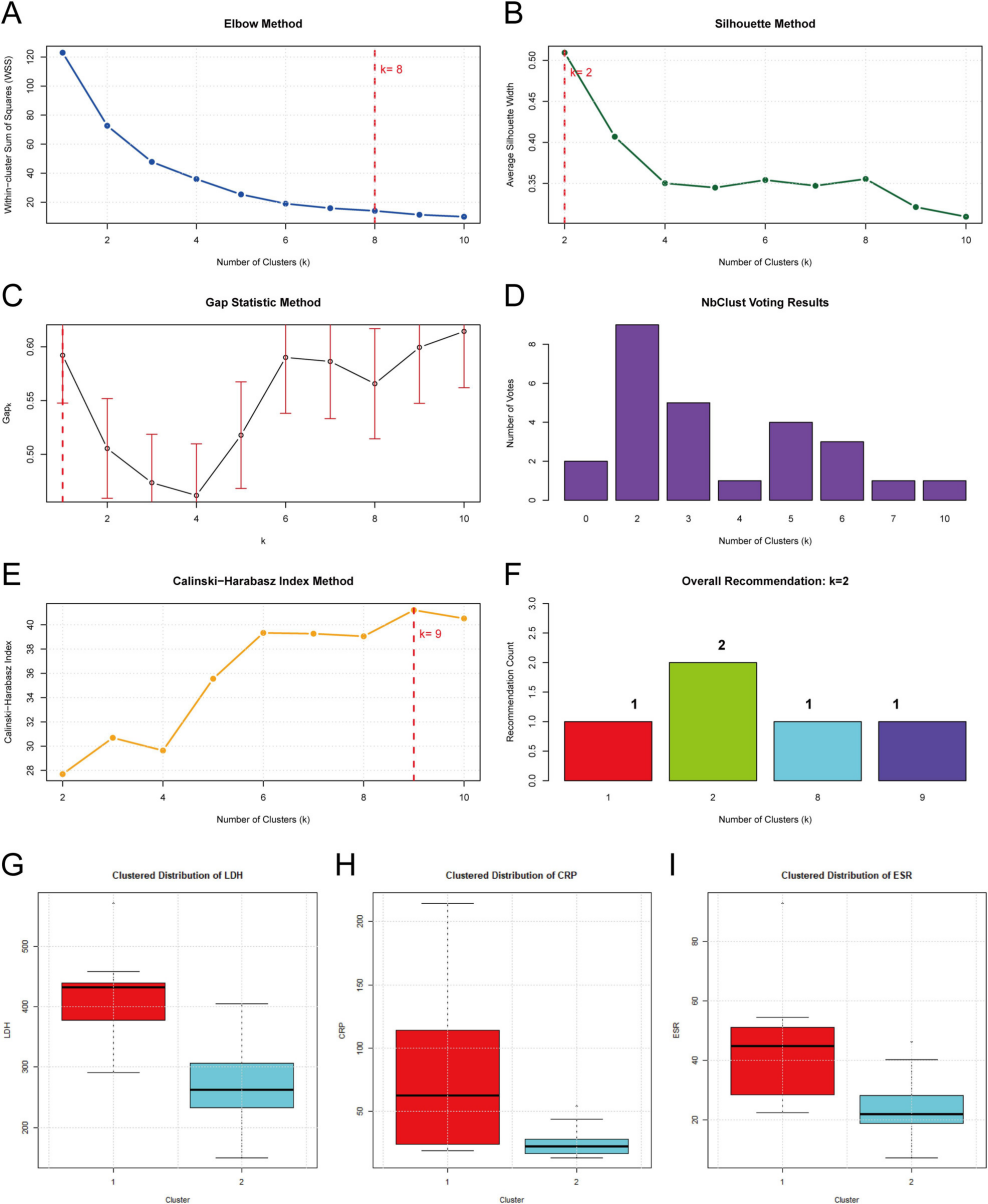
Cluster number determination and results. **(A–E)** Five methods for optimal cluster determination: Elbow method suggested *k* = 8, Silhouette method *k* = 2, Gap statistic *k* = 1, NbClust voting *k* = 2 (8 votes), and Calinski-Harabasz index *k* = 9. **(F)** Overall recommendation: *k* = 2 (R² = 0.409). **(G–I)** Distribution of three inflammatory markers with *k* = 2: Cluster 1 (High-inflammation) and Cluster 2 (Low-inflammation) showed significant differences in LDH, CRP, and ESR (all *P* < 0.001).

Considering clinical practice and the fact that LDH, CRP, and ESR were consistently identified by multiple feature selection algorithms as core inflammatory markers for predicting C. pneumoniae pneumonia consolidation, this study used unsupervised clustering methods to stratify patients into different inflammatory phenotype subgroups and evaluate differences in consolidation risk among subgroups. Five clustering evaluation methods were used to determine the optimal number of clusters (Figures 5A–E): both the silhouette coefficient and NbClust voting methods recommended *k* = 2 (majority consensus), the gap statistic method recommended *k* = 1, while the elbow method and Calinski-Harabasz index recommended *k* = 8 and *k* = 9, respectively. Considering method consistency (2/5 methods recommended *k* = 2) and clinical interpretability, *k* = 2 was finally determined as the optimal number of clusters (R² = 0.409) (Figure 5F). Detailed data are shown in Supplementary Tables S12, S13.

K-means clustering divided patients into two groups. The high-inflammation group (Cluster 1) included 9 cases (21.4%), with median (interquartile range) LDH, CRP, and ESR levels of 431.9 (377.5, 438.8) U/L, 62.8 (24.2, 114.1) mg/L, and 44.8 (28.5, 51.2) mm/h, respectively. The low-inflammation group (Cluster 2) included 33 cases (78.6%), with median (interquartile range) LDH, CRP, and ESR levels of 261.9 (232.4, 306.7) U/L, 22.5 (17.0, 27.9) mg/L, and 22.0 (18.9, 28.2) mm/h, respectively, showing statistically significant differences (*P* < 0.001) (Table 2) (Figures 5G–I).

**Table 2:** Cluster characteristics [median (IQR)].

Cluster	N	Percentage	LDH (U/L)	CRP (mg/L)	ESR (mm/h)	Consolidation Rate
1	9	21.4%	431.9 (377.5–438.8)	62.8 (24.2–114.1)	44.8 (28.5–51.2)	9/9 (100.0%)
2	33	78.6%	261.9 (232.4–306.7)	22.5 (17.0–27.9)	22.0 (18.9–28.2)	17/33 (51.5%)
*P-value*	-	-	<0.001	0.004	<0.001	0.008

Data are presented as median [interquartile range (IQR)] unless otherwise specified. LDH, lactate dehydrogenase; CRP, C-reactive protein; ESR, erythrocyte sedimentation rate. *P*-values for continuous variables were calculated using the Mann–Whitney U test; consolidation rate was compared using Fisher's exact test (*P* = 0.008). Chi-square test yielded consistent results (*χ*^2^ = 5.143, *P* = 0.023). Statistical significance was set at *P* < 0.05.

**Figure 6:**
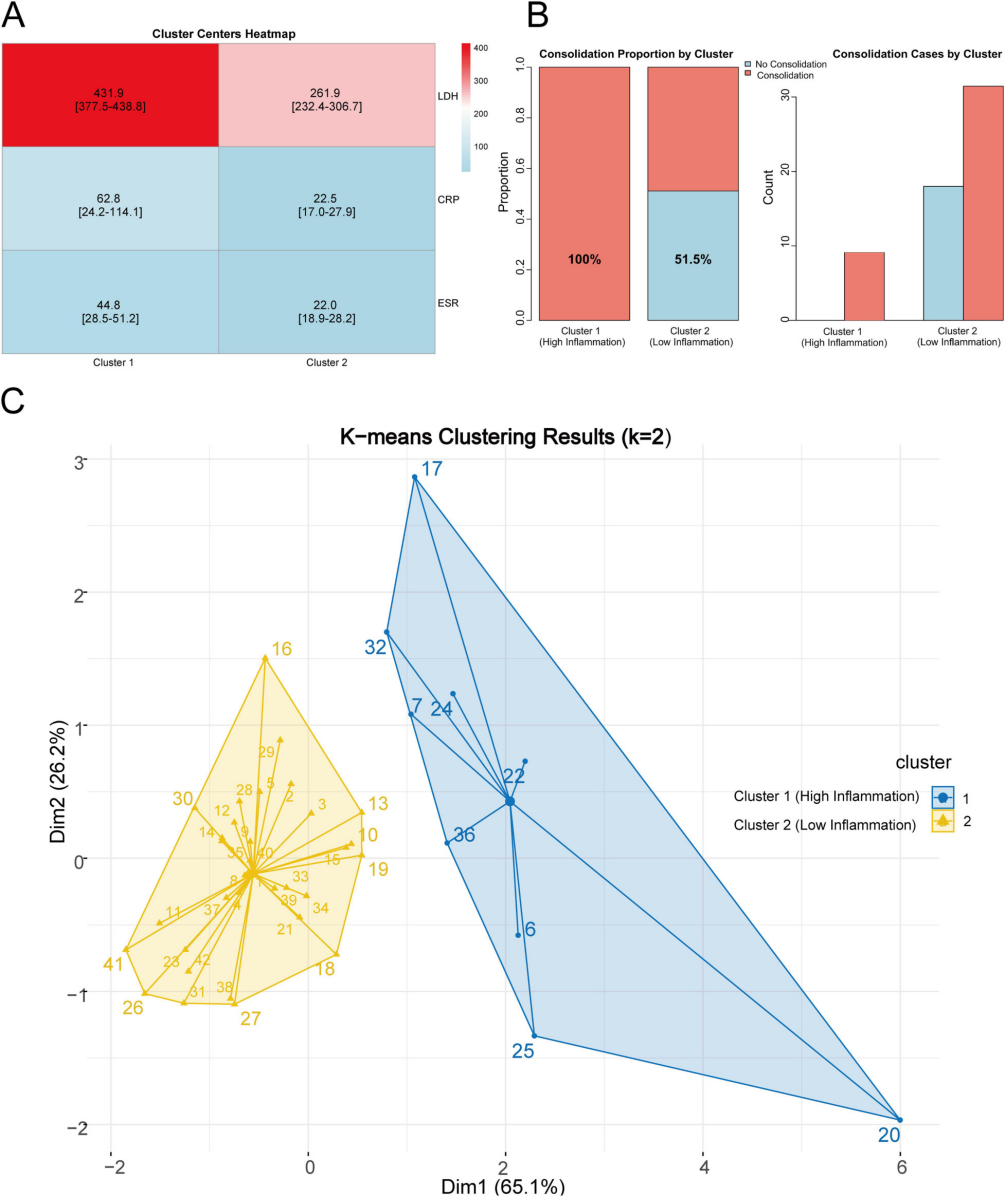
K-means Clustering analysis and risk stratification. **(A)** Cluster centers heatmap showing significant differences in LDH, CRP, and ESR between Cluster 1 (High-inflammation) and Cluster 2 (Low-inflammation) (all *P* < 0.001). **(B)** Consolidation rate comparison: Cluster 1 had 100% (9/9) and Cluster 2 had 51.5% (17/33), with statistically significant difference (*P* = 0.008). **(C)** PCA visualization (*k* = 2) demonstrating clear spatial separation between two clusters, with Dim1 and Dim2 explaining 65.1% and 26.2% of variance, respectively.

The cluster center heatmap showed clear stratification of inflammatory marker levels between the two groups (Figure 6A). The consolidation rate in Cluster 1 was significantly higher than in Cluster 2 (100% vs. 51.5%, *P* = 0.008) (Figure 6B, Table 2). The relative risk (RR) of consolidation was 0.515, indicating that the low-inflammation group has approximately half the risk of consolidation compared to the high-inflammation group. Principal component analysis visualization showed a clear separation between the two clusters in two-dimensional space, with Dim 1 explaining 65.1% of the variance and Dim 2 explaining 26.2% of the variance (Figure 6C).

### 3.5 Risk assessment system construction

To improve the clinical practicality of the risk stratification system, we developed an online risk assessment calculator based on HTML5 technology. We calculated standardized scores (Z-scores) for LDH, CRP, and ESR in 42 patients using the median and interquartile range of the Cluster 2 low-risk group as reference standards for robust standardization; the calculation formulas are shown in Table 3. The sum of these Z-scores was taken as the comprehensive score for clustering-based risk prediction. To evaluate the predictive performance of the comprehensive score, ROC curve analysis was performed. The optimal threshold was determined to be 3.75 using the Youden index (sensitivity + specificity−1), achieving a specificity of 93.9% and sensitivity of 88.9% for classifying patients with scores exceeding this threshold as Cluster 1 (Table 4).

**Table 3:** Robust standardization of indicators with cluster 2 as reference.

Variable	Median	IQR	Standardization Formula
LDH (U/L)	261.9	74.3	(LDH—261.9)/74.3
CRP (mg/L)	22.5	10.9	(CRP—22.5)/10.9
ESR (mm/h)	22.0	9.3	(ESR—22.0)/9.3

IQR, interquartile range.

**Table 4:** Diagnostic performance metrics at optimal thresholds.

Threshold	Sensitivity	Specificity	PPV	NPV	Accuracy
3.75 (Optimal)	0.889	0.939	0.800	0.969	0.929

Optimal threshold determined by Youden's Index (Sensitivity + Specificity—1). PPV, positive predictive value; NPV, negative predictive value. The area under ROC curve (AUC) = 0.993 (95% CI: 0.966–1.000).

Bootstrap validation (1,000 iterations) confirmed the stability of both the clustering solution and the risk assessment system. Cluster assignments were stable for 91.2% of patients across bootstrap resampling iterations. The bootstrap-estimated AUC was 0.949 (95% CI: 0.881–0.995), with sensitivity 95% CI of 70.0%–100.0% and specificity 95% CI of 76.5%–100.0% (Table 5), indicating that the predictive performance was robust to sampling variation and was not attributable to overfitting in this small-sample setting.

**Table 5:** Bootstrap resampling validation results (*n* = 1,000 iterations).

Metric	Original Estimate	Bootstrap Mean	95% CI
Cluster stability	—	91.2%	—
AUC	0.911	0.949	0.881–0.995
Sensitivity	84.6%	—	70.0%—100.0%
Specificity	93.8%	—	76.5%—100.0%

AUC, area under the receiver operating characteristic curve. 95% CI estimated using the bias-corrected and accelerated (BCa) bootstrap method. Cluster stability reflects the proportion of patients consistently assigned to the same cluster across 1,000 bootstrap iterations.

Sensitivity analyses further supported the robustness of the results. LOOCV demonstrated stable cluster assignments in 40 of 42 iterations (95.2%), with only 2 borderline patients reassigned upon individual exclusion. Random seed analysis confirmed that all 100 tested seeds (seeds 1–100) yielded an identical two-cluster solution (100% consistency), demonstrating that the clustering structure reflects genuine data-driven patterns rather than initialization artifacts. Threshold sensitivity analysis showed that classification performance remained acceptable (sensitivity ≥ 73.1%, specificity ≥ 81.2%) across a ±0.5 range around the Youden-optimal threshold (Table 6, Supplementary Table S14).

**Table 6:** Threshold sensitivity analysis of the composite risk score.

Threshold	Sensitivity	Specificity	PPV	NPV
−0.50 (optimal—1.0)	96.2%	50.0%	75.8%	88.9%
0.00 (optimal—0.5)	92.3%	81.2%	88.9%	86.7%
**0.50 (Youden optimal)** *	84.6%	93.8%	95.7%	78.9%
1.00 (optimal + 0.5)	73.1%	93.8%	95.0%	68.2%
1.50 (optimal + 1.0)	61.5%	100.0%	100.0%	61.5%

* Youden-optimal threshold selected for the final risk calculator (corresponding to the original manuscript threshold of 3.75 in the non-standardized score scale). PPV, positive predictive value; NPV, negative predictive value.

**Figure 7:**
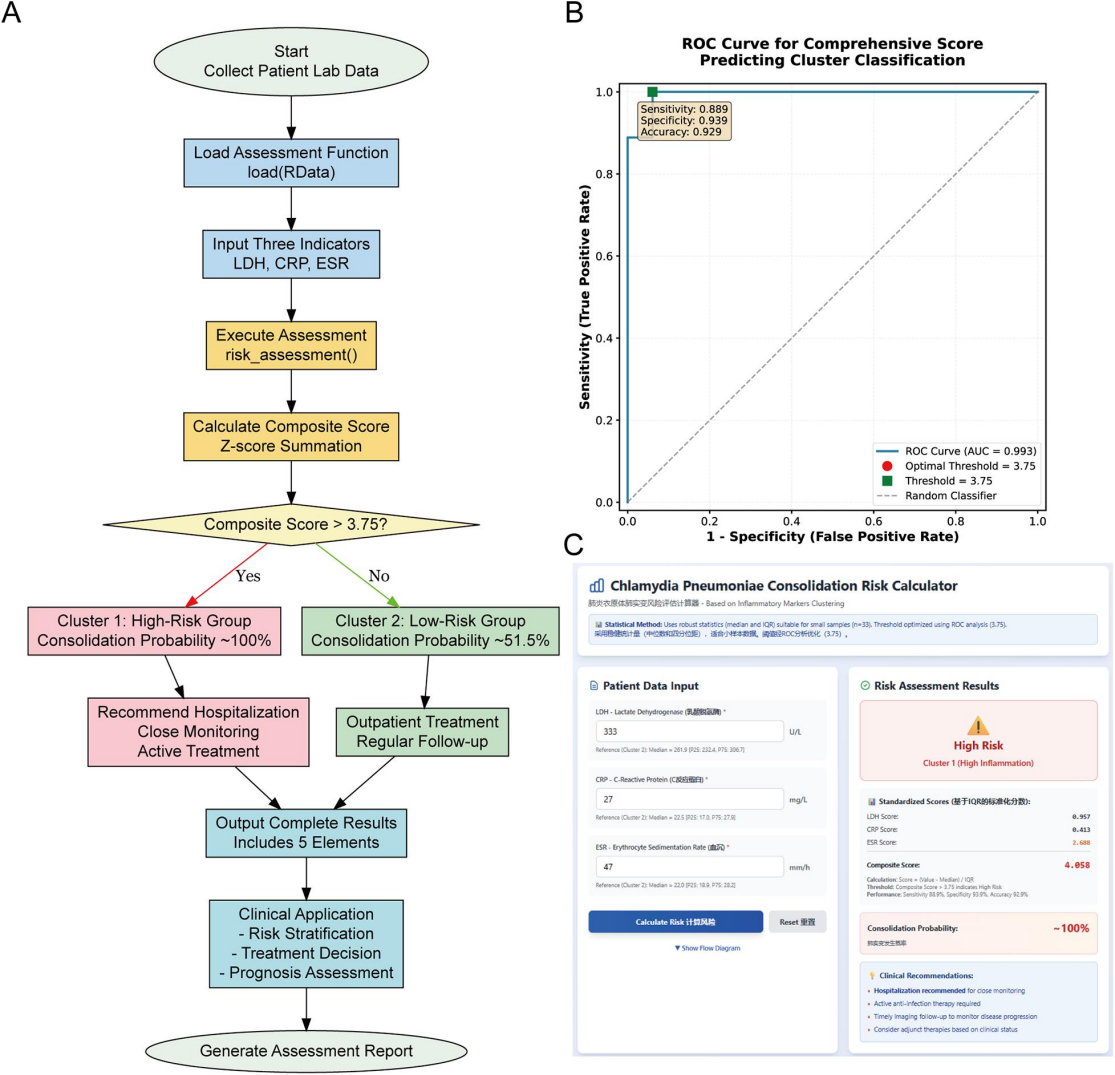
Risk assessment system for pulmonary consolidation. **(A)** Workflow from data collection to clinical decision. **(B)** ROC curve analysis (AUC = 0.993) with optimal threshold 3.75 (sensitivity: 88.9%, specificity: 93.9%). **(C)** Web-based calculator interface.

The risk assessment workflow demonstrates the complete pathway from data collection to clinical application (Figure 7A). The system first loads the assessment function and reference values; then it inputs the patient's three indicators—LDH, CRP, and ESR—calculates their Z-scores, and sums them to obtain the comprehensive score. Next, it determines the risk level based on the score threshold of 3.75 (Figure 7B), outputs complete five-item assessment results (cluster grouping, risk level, consolidation probability, comprehensive score, clinical recommendations), and finally generates an assessment report to assist clinical decision-making. The assessment operation interface is shown in Figure 7C. Detailed operation instructions for the web calculator are provided in Supplementary Material S1.

## 4 Discussion

This study systematically identified core inflammatory markers for pulmonary consolidation resulting from Chlamydia pneumoniae infection in children by integrating multiple machine learning algorithms and unsupervised clustering analysis. Additionally, it constructed an efficient risk assessment system. The results showed that LDH, CRP, and ESR are key indicators for predicting pulmonary consolidation, and the consolidation rate in children with a high inflammatory phenotype group was significantly higher, at 100% vs. 51.5%. The online risk calculator, developed using these three indicators, demonstrated excellent predictive performance (AUC = 0.993), providing a practical decision support tool for early clinical identification of high-risk patients. This study not only enriches the theoretical foundation for assessing the severity of C. pneumoniae infection in children, but also provides new insights for optimizing clinical treatment decisions and improving patient prognosis (1).

### 4.1 Methodological innovation and feasibility

The multi-algorithm ensemble feature selection strategy adopted in this study represents methodological innovation in small-sample medical research. In recent years, the application of machine learning in pediatric pneumonia prediction has become increasingly widespread, and researchers generally compare multiple algorithms to screen for optimal models (7, 11, 13). This study simultaneously applied five algorithms, including LASSO regression, SVM-RFE, Random Forest, XGBoost, and LightGBM, for feature screening. Different algorithms evaluate variable importance from different perspectives: LASSO performs feature selection via L1 regularization, SVM-RFE performs recursive elimination based on support vector machines, and tree-based models (Random Forest, XGBoost, LightGBM) evaluate feature importance based on split gain. This multi-algorithm consensus validation strategy significantly improves the reliability of feature selection and is consistent with the methodological design concepts in the pediatric severe pneumonia prediction studies by Shen et al. (8) and Zhang et al. (14). Although neither study explicitly used the exact same combination of algorithms, both involved the application of multiple machine learning algorithms in feature screening and importance assessment. In addition to feature selection, particularly for small-sample data (*n* = 42), this study used median and IQR to describe non-normally distributed variables, which is less sensitive to extreme values, compared with mean standardization methods. This approach of selecting robust statistical methods based on data characteristics is consistent with the practice of applying different statistical methods according to variable distribution types in the multicenter severe pneumonia study by Yang et al. (11). To further address the inherent limitations of small-sample analyses, bootstrap resampling (*n* = 1,000) and multiple sensitivity analyses were performed, yielding consistent results: cluster stability was 91.2%, bootstrap-estimated AUC was 0.949 (95% CI: 0.881–0.995), LOOCV confirmed stable cluster assignments in 95.2% of iterations, and random seed testing across 100 seeds yielded an identical two-cluster solution in all runs (100%). These findings collectively provide methodological validation for the robustness of the identified inflammatory phenotypes and the derived risk thresholds, supporting the reliability of the consensus feature selection approach despite the limited sample size. Furthermore, SHAP analysis provided qualitative, patient-level interpretability for the XGBoost model, directly addressing the “black-box” limitation inherent in tree-based ensemble methods. The risk thresholds derived from SHAP dependence plots showed strong concordance with the Cluster-2 low-inflammation medians, indicating that the data-driven clustering boundaries and the model's internal decision logic converge on the same biological thresholds. This cross-validation between unsupervised clustering and supervised SHAP analysis strengthens confidence in the identified markers despite the small sample size.

K-means clustering analysis, as an unsupervised learning method, can discover intrinsic stratification patterns in patients without relying on preset labels. Alexander et al. (15) confirmed in Alzheimer's disease research that K-means performed most robustly in identifying clinically actionable subgroups. This study determined the optimal number of clusters to be 2 using five methods: the elbow method, silhouette coefficient method, gap statistic, NbClust voting method, and Calinski-Harabasz index, which enhances the credibility of clustering results through a multi-indicator comprehensive evaluation. Based on the clustering results, a risk assessment system was constructed that used the Youden index to optimize the threshold. This system achieved a balanced performance of 88.9% sensitivity and 93.9% specificity in internal validation, suggesting that patient subgroups discovered through data-driven methods can be used for risk stratification. Recent studies have reported AUC values around 0.84 for pediatric pneumonia severity prediction models based on clinical variables (16). Although outcome definitions and evaluation metrics differ, the overall predictive performance of the model in this study is at a similar level to these contemporary tools.

### 4.2 International comparison of results and clinical significance

The importance of the three core inflammatory markers (LDH, CRP, ESR), identified in this study, in assessing pediatric pneumonia severity has been widely confirmed by international studies. While LDH, CRP, and ESR are non-specific inflammatory markers common across various pneumonia etiologies, their combined elevation pattern and consensus identification by multiple independent machine learning algorithms suggest that these markers collectively capture the inflammatory burden associated with pulmonary consolidation in C. pneumoniae infection. Recent meta-analyses have confirmed that elevated serum LDH is significantly associated with pneumonia severity in children (Standardized Mean Difference = 0.51, 95% CI: 0.36–0.66) (17) and serves as an independent predictor of refractory pneumonia (18, 19). Similarly, CRP has been identified as a significant risk factor for lobar pneumonia in pediatric machine learning models (8). A recent comprehensive review further supports the prognostic value of combining CRP, LDH, and other inflammatory markers in assessing pediatric pneumonia severity (10). The clinical value of these biomarkers lies not in their specificity to a single pathogen, but in their ability to reflect the severity of the inflammatory cascade that drives tissue consolidation, regardless of the underlying etiology. This is consistent with the multi-algorithm consensus approach used in this study, where the convergent identification of these markers by five distinct algorithms strengthens the robustness of these findings despite their non-specific nature. Notably, relatively few studies have examined inflammatory markers specific to C. pneumoniae infection. Liu et al. (4), in a study anticipated to be published in 2025, found that pulmonary consolidation and hypoxemia were the most common respiratory complications in children with C. pneumoniae infection, which aligns with the observed higher incidence of hypoxemia in the consolidation group in this study, although this difference did not reach statistical significance. Furthermore, this study found that the duration of disease before admission in the consolidation group was significantly longer than in the non-consolidation group (10 days vs. 6 days). This finding suggests that prolonged disease duration may reflect the process of disease progression and sustained inflammatory response, consistent with the clinical observation of a “prolonged disease course” in M. pneumoniae infection mentioned in the review by Yan et al. (20).

The consolidation group in this study showed significantly elevated white blood cell count, neutrophil percentage, and NLR, along with decreased lymphocyte percentage. This inflammatory cell pattern is consistent with previous research findings on severe Mycoplasma pneumoniae pneumonia (MPP) in children (21, 22). This suggests that when pulmonary consolidation, a serious pulmonary complication, occurs, the immune response in children may exhibit certain common characteristics in peripheral blood cell distribution. However, no significant difference in PCT levels was observed between the two groups, which is consistent with recent evidence that procalcitonin has only moderate accuracy for distinguishing typical from atypical lower respiratory pathogens, including atypical organisms such as Chlamydia and Mycoplasma (6). These findings suggest that PCT primarily reflects systemic inflammatory responses associated with typical bacterial infections and has limited utility in differentiating infections caused by atypical pathogens. In line with this, systematic review data indicate that PCT remains a useful biomarker for identifying severe bacterial infections, including sepsis, when interpreted alongside clinical assessment (23). The lack of significant PCT elevation in C. pneumoniae infection may be attributed to its intracellular parasitic nature. PCT production is primarily induced by circulating bacterial endotoxins (lipopolysaccharides) and pro-inflammatory cytokines such as IL-1β, IL-6, and TNF-α, whereas interferon-γ (IFN-γ) suppresses CALC-1 gene expression, thereby reducing PCT synthesis (24, 25). As an obligate intracellular pathogen, C. pneumoniae does not release substantial endotoxin into the bloodstream. Instead, it primarily triggers cell-mediated immune responses through Toll-like receptor 2 (TLR2)- and MyD88-dependent pathways, leading to the activation of Th1-type CD4+ T cells and the production of IFN-γ and TNF-α (26, 27). This Th1-dominant cytokine milieu, particularly the elevated IFN-γ levels, may in turn down-regulate PCT production. This pattern is consistent with observations in other atypical pathogen infections, where Mycoplasma and Chlamydia species typically elicit inflammatory profiles characterized by relatively preserved PCT levels despite active systemic inflammation reflected by elevated CRP and ESR.

### 4.3 Pathophysiological mechanisms of inflammatory marker elevation

The elevated inflammatory marker pattern observed in this study reflects the pathophysiological process of pulmonary involvement caused by C. pneumoniae infection. As an obligate intracellular pathogen, Chlamydia pneumoniae undergoes a biphasic developmental cycle in which elementary bodies (EBs) enter respiratory epithelial cells via receptor-mediated endocytosis and differentiate into reticulate bodies (RBs) that replicate intracellularly (28). This intracellular replication activates innate immune signaling pathways, leading to increased production of pro-inflammatory cytokines such as IL-6, IL-8, and TNF-α from infected epithelial cells and macrophages (29). These cytokines promote recruitment of neutrophils and monocytes, resulting in a predominantly neutrophil-driven early inflammatory response (30).

Lactate dehydrogenase is an important marker of tissue damage and cell lysis. Its release mechanism is universal: in severe pulmonary infections, sustained inflammatory responses lead to the disruption of the integrity of lung tissue cell membranes, causing intracellular LDH to enter the bloodstream. This LDH release mechanism has been fully confirmed in infections with pathogens such as M. pneumoniae (31). Gupta et al. (32) pointed out that LDH has a dual role in inflammatory diseases: it serves both as an inflammatory marker and as a metabolic marker. Moreover, the LDH level is closely related to disease severity. In this study, the median LDH level in the consolidation group (patients exhibiting lung tissue consolidation) reached 319.66 U/L, which was significantly higher than the 218.62 U/L observed in the non-consolidation group, which includes patients without such consolidation. This difference may reflect more extensive tissue damage and inflammatory cell infiltration in the lung tissue consolidation area. CRP, an acute-phase protein, is synthesized by the liver under the stimulation of pro-inflammatory factors such as IL-6 (33). This study found significantly elevated CRP levels in the consolidation group, which was consistent with increased IL-6 levels. An elevated ESR reflects increased plasma fibrinogen and altered red blood cell surface charge caused by inflammation (34). It is worth noting that all three indicators in the high-inflammation group (Cluster 1) were significantly higher than those in the low-inflammation group. Moreover, the percentage of patients showing consolidation on imaging reached 100%; this suggests a threshold effect in the amplification of the inflammatory cascade. Specifically, when the inflammatory response exceeds a certain threshold, the risk of alveolar exudation and consolidation increases sharply. This finding provides a new perspective for understanding the immunopathological mechanisms of C. pneumoniae infection.

### 4.4 Clinical application value and practical significance

The risk assessment system developed in this study has significant clinical application value. First, in terms of accessibility, the system only requires the following three routine laboratory test indicators: LDH, CRP, and ESR. These indicators can be obtained at primary care facilities at low testing costs, and results are available in a short time, which provides the system with good clinical accessibility (35). Second, regarding convenience, the online calculator based on HTML5 technology enables rapid bedside assessment. Physicians can immediately obtain risk stratification results when children visit the clinic, without complex calculations or special equipment (36). Such immediacy is crucial for guiding early treatment decisions, especially in identifying high-risk children who need more aggressive intervention.

From a clinical decision-making perspective, identifying high-risk children (comprehensive score >3.75) helps optimize medical resource allocation. For these children, clinicians may consider early initiation of corticosteroid therapy to control excessive inflammatory responses. This study found a significantly longer duration of corticosteroid use in the consolidation group. This finding is consistent with clinical practice, where severe cases require more intensive anti-inflammatory management strategies. The efficacy of such strategies in severe pneumonia has been supported by a recent systematic review and meta-analysis (37).In addition, for patients with pulmonary consolidation showing poor treatment response, timely use of flexible fiber-optic bronchoscopy for intervention is one of the feasible management options (38).

Furthermore, this risk assessment system can also be used for outpatient triage and hospitalization decisions. Low-risk children (score ≤ 3.75), are considered for outpatient treatment or observation, while high-risk children are recommended for hospitalization to receive closer monitoring and treatment. This stratified management strategy, based on objective indicators, helps reduce unnecessary hospitalizations while ensuring that children who truly need hospitalization receive timely care. This approach aligns with the principles of precision medicine and value-based care (39). In the current context of limited medical resources, such tools optimize resource allocation and have significant health economic benefits, including cost savings and improved efficiency.

### 4.5 Study limitations

This study has certain limitations. First, the sample size was small (*n* = 42) and it was a single-center retrospective study, which may limit the generalizability of the results. Potential confounders, including age, gender, and comorbidities, were assessed through univariate analysis. While no significant differences were found between groups for most demographic and clinical characteristics (Supplementary Table S1), the retrospective design limits our ability to fully control for unmeasured confounders. Future prospective studies should consider multivariable adjustment or propensity score matching to better account for potential confounding. Moreover, the 100% consolidation rate observed in the high-inflammation cluster (Cluster 1) should be interpreted with caution. This finding may partly reflect selection bias inherent in the retrospective study design, as patients with more severe presentations (and thus higher inflammatory markers) may have been more likely to undergo detailed imaging and be included in the study. The small size of the high-inflammation group (*n* = 9) also means that the 100% rate may not be robust and could change with larger samples. Prospective studies with standardized imaging protocols are needed to confirm this association. The generalizability of the model still needs further validation in larger sample sizes and multicenter prospective studies. External validation on an independent cohort was not feasible in the current study due to the single-center design and the limited availability of comparable C. pneumoniae-specific pediatric datasets. Bootstrap resampling (1,000 iterations) was therefore employed as the primary internal validation strategy, which is the recommended approach for small-sample clinical prediction research and has been widely applied in analogous contexts. The convergent findings across bootstrap validation, LOOCV, and seed sensitivity analyses collectively support the stability of the reported results. Nonetheless, external prospective validation in multicenter cohorts remains a priority, and we plan to collaborate with regional pediatric centers to establish an external validation dataset. Second, the study only included children with single-pathogen C. pneumoniae infection. Although this improved internal validity, the model performance in the clinically common mixed infection scenario has yet to be evaluated. Third, in addition to pathogen considerations, this study was based only on laboratory indicators at admission and did not include dynamic monitoring data. Future studies can develop dynamic prediction models using indicators measured at multiple time points to better characterize disease progression. In addition, potential influencing factors such as genetic susceptibility and immune function status, were not included, which may limit the comprehensiveness of individualized risk assessment. Finally, although the risk calculator demonstrated good predictive performance, its clinical application value, and actual effect on improving prognosis still need further verification through interventional studies.

## 5 Conclusions

This study consistently identified LDH, CRP, and ESR as core inflammatory markers for predicting pulmonary consolidation in children with C. pneumoniae infection through multiple machine learning algorithms. It also established an efficient risk assessment system based on K-means clustering analysis. The system uses routine laboratory indicators and has good clinical accessibility and utility. It can be used for early identification of high-risk children to guide individualized treatment decisions. To further validate and extend these findings, multicenter prospective studies with larger sample sizes are warranted. We are currently exploring collaborative opportunities with other pediatric centers in the region to establish a multicenter validation cohort. Future research should also investigate the integration of dynamic monitoring data (e.g., serial laboratory measurements) and additional biomarkers to enhance the predictive accuracy and clinical utility of the risk assessment system.

## Data Availability

The original contributions presented in the study are included in the article/Supplementary Material, further inquiries can be directed to the corresponding author.
