# Supplementary material for: Machine learning-based identification of inflammatory biomarkers for predicting pulmonary consolidation in children with Chlamydia pneumoniae infection
Source: Front Pediatr. 2026 May 4;14:1779116. doi: 10.3389/fped.2026.1779116 (PMC13180937; doi:10.3389/fped.2026.1779116)
Supplement: Supplementary file 1 [file Datasheet1.zip › Supplementary Material S1/CP_Risk_Calculator.html]

CP Consolidation Risk Calculator | 肺炎衣原体肺实变风险评估
