# Supplementary material for: Machine learning-based identification of inflammatory biomarkers for predicting pulmonary consolidation in children with Chlamydia pneumoniae infection
Source: Front Pediatr. 2026 May 4;14:1779116. doi: 10.3389/fped.2026.1779116 (PMC13180937; doi:10.3389/fped.2026.1779116)
Supplement: Supplementary file 1 [file Datasheet1.zip › Supplementary Material S1/User_Manual_English.pdf]

CP Consolidation Risk Calculator

User Manual

Overview

This calculator predicts pulmonary consolidation risk in Chlamydia pneumoniae pneumonia patients using inflammatory marker clustering analysis. It employs robust statistics (median and IQR) optimized for small sample sizes.

How to Use

Step-by-Step Instructions

1. Open the calculator in your web browser
2. Enter the patient's laboratory values:

• LDH (Lactate Dehydrogenase) in U/L

• CRP (C-Reactive Protein) in mg/L

• ESR (Erythrocyte Sedimentation Rate) in mm/h

3. Click the 'Calculate Risk' button
4. Review the results showing risk classification and consolidation probability
5. Optional: Click 'Show Decision Flow' to view the calculation process

Understanding Results

Risk Classifications

Classification	Composite Score	Consolidation Risk
High Risk (Cluster 1)	> 3.75	~100%
Low Risk (Cluster 2)	≤ 3.75	~51.5%

Calculation Method

The calculator uses robust standardization based on Cluster 2 reference values:

$$\text{Score} = (\text{Value} - \text{Median}) / \text{IQR}$$

Where:

• Median = reference median from Cluster 2 (low-risk group)

• IQR = Interquartile Range (Q3 - Q1)

The composite score is the sum of individual scores for LDH, CRP, and ESR. A threshold of 3.75 was optimized using ROC curve analysis with Youden's Index.

Performance Metrics

• Sensitivity: 88.9% • Specificity: 93.9% • Accuracy: 92.9% • AUC: 0.993

Important Notes

This tool is for research and educational purposes only

- Clinical decisions should be made by qualified healthcare professionals
- Consider all patient information including imaging and clinical symptoms
- Patients with scores near threshold (3.75) require close monitoring
- Based on K-means clustering analysis of 42 patients

For questions or feedback, please contact your healthcare provider
