# Supplementary material for: Machine learning-based identification of inflammatory biomarkers for predicting pulmonary consolidation in children with Chlamydia pneumoniae infection
Source: Front Pediatr. 2026 May 4;14:1779116. doi: 10.3389/fped.2026.1779116 (PMC13180937; doi:10.3389/fped.2026.1779116)
Supplement: Supplementary file 2 [file Table1.docx]

Supplementary Tables

**Supplementary Table S1. Complete List of 42 Candidate Variables Included in the Analysis**

| **No.** | **Variable** | **Type** | **Unit** | **Description** |
| --- | --- | --- | --- | --- |
| ***Demographics*** | | | | |
| 1 | Gender | Categorical (Binary) | — | Male or female, n (%) |
| 2 | Age | Continuous | Years | Age at admission, median (IQR) |
| ***Clinical Characteristics*** | | | | |
| 3 | Duration of illness on admission | Continuous | Days | Days from symptom onset to hospital admission, median (IQR) |
| 4 | History of food/drug allergy | Categorical (Binary) | — | Documented history of food or drug allergy, n (%) |
| 5 | Cough characteristics | Categorical (Nominal) | — | Classified as: no cough, dry cough, wet cough, or dry-to-wet cough, n (%) |
| 6 | Hypoxemia | Categorical (Binary) | — | Presence of hypoxemia at admission, n (%) |
| 7 | Fever | Categorical (Binary) | — | Presence of fever at admission, n (%) |
| ***Pathogen Detection*** | | | | |
| 8 | C. pneumoniae DNA copy number | Continuous | Copies | Quantitative PCR-detected C. pneumoniae nucleic acid copy number, median (IQR) |
| 9 | Estimated C. pneumoniae concentration | Continuous | Copies/mL | Estimated C. pneumoniae DNA concentration calculated from standard curve, median (IQR) |
| ***Laboratory Findings – Blood Count*** | | | | |
| 10 | White blood cell count (WBC) | Continuous | ×10⁹/L | Total white blood cell count, median (IQR) |
| 11 | Neutrophil percentage | Continuous | % | Proportion of neutrophils in peripheral blood, median (IQR) |
| 12 | Neutrophil count | Continuous | ×10⁹/L | Absolute neutrophil count, median (IQR) |
| 13 | Lymphocyte percentage | Continuous | % | Proportion of lymphocytes in peripheral blood, median (IQR) |
| 14 | Lymphocyte count | Continuous | ×10⁹/L | Absolute lymphocyte count, median (IQR) |
| 15 | Neutrophil-to-lymphocyte ratio (NLR) | Continuous | Ratio | Calculated as neutrophil count / lymphocyte count, median (IQR) |
| 16 | Eosinophil percentage | Continuous | % | Proportion of eosinophils in peripheral blood, median (IQR) |
| 17 | Eosinophil count | Continuous | ×10⁹/L | Absolute eosinophil count, median (IQR) |
| 18 | Hemoglobin | Continuous | g/L | Hemoglobin concentration, median (IQR) |
| 19 | Platelet count | Continuous | ×10⁹/L | Total platelet count, median (IQR) |
| ***Laboratory Findings – Inflammatory/Biochemical*** | | | | |
| 20 | C-reactive protein (CRP) | Continuous | mg/L | Serum C-reactive protein level, median (IQR) |
| 21 | Interleukin-6 (IL-6) | Continuous | pg/mL | Serum interleukin-6 level, median (IQR) |
| 22 | Procalcitonin (PCT) | Continuous | ng/mL | Serum procalcitonin level, median (IQR) |
| 23 | Serum amylase | Continuous | U/L | Serum amylase activity, median (IQR) |
| 24 | Erythrocyte sedimentation rate (ESR) | Continuous | mm/h | Erythrocyte sedimentation rate, median (IQR) |
| 25 | Lactate dehydrogenase (LDH) | Continuous | U/L | Serum lactate dehydrogenase level, median (IQR) |
| 26 | D-dimer | Continuous | μg/mL | Plasma D-dimer level, median (IQR) |
| 27 | Specific IgE | Continuous | IU/mL | Serum allergen-specific immunoglobulin E, median (IQR) |
| 28 | Total IgE | Continuous | IU/mL | Serum total immunoglobulin E, median (IQR) |
| ***Imaging Findings*** | | | | |
| 29 | Right upper lobe involvement | Categorical (Binary) | — | Presence of lesion in the right upper lobe on chest imaging, n (%) |
| 30 | Right middle lobe involvement | Categorical (Binary) | — | Presence of lesion in the right middle lobe on chest imaging, n (%) |
| 31 | Right lower lobe involvement | Categorical (Binary) | — | Presence of lesion in the right lower lobe on chest imaging, n (%) |
| 32 | Left upper lobe involvement | Categorical (Binary) | — | Presence of lesion in the left upper lobe on chest imaging, n (%) |
| 33 | Left lower lobe involvement | Categorical (Binary) | — | Presence of lesion in the left lower lobe on chest imaging, n (%) |
| 34 | Number of lobes involved | Continuous (Discrete) | Count | Total number of lung lobes with lesions, median (IQR) |
| 35 | Bilateral lung involvement | Categorical (Binary) | — | Lesions present in both lungs, n (%) |
| 36 | Ground-glass opacity | Categorical (Binary) | — | Presence of ground-glass opacity on chest imaging, n (%) |
| 37 | Nodule | Categorical (Binary) | — | Presence of pulmonary nodules on chest imaging, n (%) |
| 38 | Patchy shadow | Categorical (Binary) | — | Presence of patchy shadows on chest imaging, n (%) |
| 39 | Air bronchogram | Categorical (Binary) | — | Presence of air bronchogram sign on chest imaging, n (%) |
| ***Complications and Comorbidities*** | | | | |
| 40 | Extrapulmonary symptoms | Categorical (Binary) | — | Presence of extrapulmonary manifestations, n (%) |
| 41 | Pleural effusion | Categorical (Binary) | — | Presence of pleural effusion on chest imaging, n (%) |
| 42 | Allergic rhinitis | Categorical (Binary) | — | History of allergic rhinitis, n (%) |

Note: A total of 42 variables were retained after excluding treatment-related variables from the initial 52 candidate variables. Treatment-related variables excluded from the analysis included: macrolide use, minocycline use, cephalosporin use, other antibiotic use, number of antibiotic types, corticosteroid use, corticosteroid duration, bronchoscopy performed, and specimen type (throat swab/sputum/bronchoalveolar lavage). Categorical variables are presented as n (%) and continuous variables as median (interquartile range, IQR) in baseline comparisons. Binary categorical variables were coded as 0/1 for machine learning analyses. IQR, interquartile range; WBC, white blood cell; NLR, neutrophil-to-lymphocyte ratio; CRP, C-reactive protein; IL-6, interleukin-6; PCT, procalcitonin; ESR, erythrocyte sedimentation rate; LDH, lactate dehydrogenase; IgE, immunoglobulin E; C. pneumoniae, Chlamydia pneumoniae.

**Supplementary Table S2. Hyperparameter Search Spaces, Cross-Validation Strategies, and Optimal Configurations for Five Machine Learning Algorithms**

| **Algorithm** | **Cross-ValidationStrategy** | **HyperparameterSearch Method** | **Hyperparameter Search Space** | **Optimal Values** | **Feature SelectionCriterion** | **R Package** |
| --- | --- | --- | --- | --- | --- | --- |
| LASSO Regression | 10-fold CV | Regularization path  (auto-generated) | alpha = 1 (L1 penalty); lambda sequence automatically generated by glmnet across full regularization path | lambda.min = 0.0342;  lambda.1se = 0.0951 | lambda.1se criterion  (3 variables selected) | glmnet (v4.1-8) |
| SVM-RFE | 10-fold CV (outer);  10-fold CV (inner,  within svmRFE.wrap) | Grid search  (tune function) | Kernel: linear;  Cost (C): {0.1, 1, 10, 100} | Kernel: linear;  Cost: optimized via  grid search | Recursive feature  elimination ranking  (average rank across  CV folds) | e1071 (v1.7-13),  caret |
| Random Forest | 10-fold CV | Grid search | mtry: searched over candidate values;  ntree: 500 | mtry = 5;  ntree = 500;  OOB error stabilized  at ~200 trees | Mean decrease in Gini  impurity (threshold  ≥ 2.0) | randomForest |
| XGBoost | 5-fold CV | Grid search  (caret::train) | nrounds: {100, 200};  max_depth: {3, 5, 7};  eta (learning rate): {0.01, 0.1, 0.3};  gamma: 0;  colsample_bytree: 1;  min_child_weight: 1;  subsample: 1 | Selected by best  average ROC across  validation folds  (nrounds = 100, max_depth = 3, and eta = 0.1) | Feature Gain values | xgboost,  caret |
| LightGBM | 5-fold stratified CV | Grid search  (lgb.cv) | num_leaves: {5, 10, 15};  learning_rate: {0.01, 0.03};  min_data_in_leaf: {3, 5, 8};  Fixed: feature_fraction = 0.7,  bagging_fraction = 0.7,  lambda_l1 = 0.5, lambda_l2 = 0.5,  min_gain_to_split = 0.01;  nrounds: up to 500  (early stopping = 50 rounds) | Determined by best  CV AUC across 18  parameter combinations | Gain > 0.01 or  top 15 features | lightgbm |

Note: CV, cross-validation; LASSO, least absolute shrinkage and selection operator; SVM-RFE, support vector machine-recursive feature elimination; OOB, out-of-bag; AUC, area under the receiver operating characteristic curve. All analyses were performed with a random seed of 12345 for reproducibility.

**Supplementary Table S3. Baseline Characteristics of Children with Chlamydia pneumoniae pneumonia Infection**

| **Characteristics** | **Consolidation (n=26)** | **Non-consolidation (n=16)** | **P-value** |
| --- | --- | --- | --- |
| **Demographics** | | | |
| Male, n (%) | 14 (53.85) | 9 (56.25) | 1.000 |
| Age (years), median (IQR) | 13.00 (11.00, 14.00) | 11.50 (9.00, 14.00) | 0.091 |
| ≤6 yr | 0 (0.0%) | 1 (6.3%)* | 0.261 |
| 7–11 yr | 8 (30.8%) | 7 (43.8%) |  |
| ≥12 yr | 18 (69.2%) | 8 (50.0%) |  |
| Body weight (kg) | 44.3 (35.6, 50.7) | 36.2 (29.0, 47.0) | 0.799 |
| **Clinical Characteristics** | | | |
| Duration of illness on admission (days), median (IQR) | 10.00 (6.00, 14.00) | 6.00 (5.25, 9.25) | 0.045 |
| Admission season |  |  |  |
| Spring | 3 (11.5%) | 2 (12.5%) | 0.611 |
| Summer | 2 (7.7%) | 2 (12.5%) |  |
| Autumn | 9 (34.6%) | 7 (43.8%) |  |
| Winter | 12 (46.2%) | 5 (31.3%) |  |
| History of food/drug allergy, n (%) | 5 (19.23) | 3 (18.75) | 1.000 |
| Cough characteristics |  |  |  |
| No cough, n (%) | 1 (3.85) | 0 (0.00) | 0.600 |
| Dry cough, n (%) | 7 (26.92) | 4 (25.00) |  |
| Wet cough, n (%) | 10 (38.46) | 9 (56.25) |  |
| Dry to wet cough, n (%) | 8 (30.77) | 3 (18.75) |  |
| Hypoxemia, n (%) | 9 (34.62) | 3 (18.75) | 0.451 |
| Fever, n (%) | 13 (50.00) | 10 (62.50) | 0.638 |
| **Treatment** | | | |
| Macrolides, n (%) | 3 (11.54) | 2 (12.50) | 1.000 |
| Minocycline, n (%) | 19 (73.08) | 13 (81.25) | 0.817 |
| Cephalosporins, n (%) | 11 (42.31) | 7 (43.75) | 1.000 |
| Other antibiotics, n (%) | 4 (15.38) | 2 (12.50) | 0.490 |
| Number of antibiotic types |  |  | 0.507 |
| 1 type, n (%) | 13 (50.00) | 8 (50.00) |  |
| 2 types, n (%) | 11 (42.31) | 8 (50.00) |  |
| 3 types, n (%) | 2 (7.69) | 0 (0.00) |  |
| Corticosteroid duration (d), median (IQR) | 4.00 (0.00, 5.00) | 0.00 (0.00, 4.00) | 0.022 |
| Corticosteroid use, n (%) | 18 (69.23) | 6 (37.50) | 0.090 |
| Bronchoscopy performed, n (%) | 14 (53.85) | 9 (56.25) | 1.000 |
| **Pathogen Detection** | | | |
| Sample type |  |  | 0.463 |
| Throat swab, n (%) | 9 (34.62) | 4 (25.00) |  |
| Sputum, n (%) | 4 (15.38) | 5 (31.25) |  |
| Bronchoalveolar lavage, n (%) | 13 (50.00) | 7 (43.75) |  |
| C. pneumoniae DNA copies, median (IQR) | 12808.50 (2871.50, 35463.00) | 30375.00 (5743.25, 49123.00) | 0.219 |
| C. pneumoniae concentration (copies/mL), median (IQR) | 1000000.00 (7650.00, 1000000.00) | 1000000.00 (17750.00, 1000000.00) | 0.536 |
| **Laboratory Findings** | | | |
| White blood cells (×10⁹/L), median (IQR) | 10.76 (9.93, 13.98) | 8.64 (7.60, 9.89) | 0.001 |
| Neutrophil percentage (%), median (IQR) | 79.15 (70.10, 84.16) | 60.44 (58.12, 72.58) | <0.001 |
| Neutrophil count (×10⁹/L), median (IQR) | 6.71 (6.37, 8.03) | 5.18 (4.53, 6.40) | 0.005 |
| Lymphocyte percentage (%), median (IQR) | 16.63 (13.55, 20.40) | 24.84 (16.00, 29.26) | 0.029 |
| Lymphocyte count (×10⁹/L), median (IQR) | 1.70 (1.25, 2.01) | 2.00 (1.34, 2.19) | 0.219 |
| NLR, median (IQR) | 4.69 (3.38, 6.07) | 2.43 (1.86, 4.58) | 0.008 |
| Eosinophil percentage (%), median (IQR) | 2.65 (1.02, 3.60) | 2.90 (2.42, 4.90) | 0.223 |
| Eosinophil count (×10⁹/L), median (IQR) | 0.28 (0.08, 0.40) | 0.24 (0.21, 0.39) | 0.707 |
| Hemoglobin (g/L), median (IQR) | 133.00 (128.00, 140.50) | 130.50 (125.00, 140.25) | 0.534 |
| Platelet count (×10⁹/L), median (IQR) | 343.50 (321.25, 394.50) | 296.50 (246.00, 341.75) | 0.048 |
| C-reactive protein (mg/L), median (IQR) | 26.80 (22.71, 51.38) | 17.00 (15.00, 23.25) | <0.001 |
| Interleukin-6 (pg/mL), median (IQR) | 15.37 (13.01, 24.46) | 12.41 (11.39, 15.33) | 0.045 |
| Procalcitonin (ng/mL), median (IQR) | 0.06 (0.03, 0.09) | 0.04 (0.03, 0.05) | 0.133 |
| Serum amylase (U/L), median (IQR) | 39.58 (8.54, 91.15) | 20.07 (6.70, 65.47) | 0.460 |
| ESR (mm/h), median (IQR) | 30.42 (23.05, 39.82) | 19.29 (13.58, 22.13) | <0.001 |
| LDH (U/L), median (IQR) | 319.66 (265.11, 372.41) | 218.62 (172.96, 274.34) | <0.001 |
| D-dimer (μg/mL), median (IQR) | 219.80 (167.65, 338.45) | 155.50 (123.00, 251.00) | 0.068 |
| Specific IgE (IU/mL), median (IQR) | 289.69 (197.99, 585.30) | 254.95 (172.99, 468.91) | 0.493 |
| Total IgE (IU/mL), median (IQR) | 181.99 (83.81, 516.36) | 82.30 (56.80, 186.00) | 0.095 |
| **Imaging Findings** | | | |
| Right upper lobe involvement, n (%) | 10 (40.00) | 4 (25.00) | 0.515 |
| Right middle lobe involvement, n (%) | 8 (30.77) | 4 (25.00) | 0.960 |
| Right lower lobe involvement, n (%) | 12 (46.15) | 7 (43.75) | 1.000 |
| Left upper lobe involvement, n (%) | 6 (23.08) | 1 (6.25) | 0.320 |
| Left lower lobe involvement, n (%) | 9 (34.62) | 3 (18.75) | 0.451 |
| Number of lobes involved, median (IQR) | 1.00 (1.00, 2.00) | 1.00 (1.00, 1.00) | 0.078 |
| Bilateral lung involvement, n (%) | 6 (23.08) | 1 (6.25) | 0.320 |
| Ground-glass opacity, n (%) | 4 (15.38) | 0 (0.00) | 0.280 |
| Nodules, n (%) | 11 (42.31) | 7 (43.75) | 1.000 |
| Patchy shadows, n (%) | 14 (53.85) | 10 (62.50) | 0.819 |
| Air bronchogram sign, n (%) | 4 (15.38) | 7 (43.75) | 0.095 |
| **Complications and Comorbidities** | | | |
| Extrapulmonary symptoms, n (%) | 8 (30.77) | 5 (31.25) | 1.000 |
| Pleural effusion, n (%) | 2 (7.69) | 0 (0.00) | 0.517 |
| Allergic rhinitis, n (%) | 12 (46.15) | 4 (25.00) | 0.297 |

Note: Data are presented as n (%) for categorical variables and median (IQR) for continuous variables. IQR, interquartile range; NLR, neutrophil-to-lymphocyte ratio; ESR, erythrocyte sedimentation rate; LDH, lactate dehydrogenase; IgE, immunoglobulin E. P-values were calculated using Chi-square test or Fisher's exact test for categorical variables and Mann-Whitney U test for continuous variables.

**Supplementary Table S4. Univariate Logistic Regression Analysis of Risk Factors for Pulmonary Consolidation in Chlamydophila pneumoniae Infection**

| **Variable** | **OR** | **95% CI** | **P-value** |
| --- | --- | --- | --- |
| Male | 1.1777 | 0.6414-2.1624 | 0.598 |
| Age (years) | 1.1121 | 0.9886-1.2511 | 0.077 |
| Duration of illness on admission (days) | 1.1578 | 1.0993-1.2194 | **< 0.001** |
| History of food/drug allergy | 1.0526 | 0.4873-2.2737 | 0.896 |
| Cough characteristics | 1.1093 | 0.7547-1.6304 | 0.598 |
| Hypoxemia | 1.7507 | 0.8963-3.4194 | 0.101 |
| Fever | 0.7667 | 0.4176-1.4077 | 0.392 |
| Chlamydophila pneumoniae nucleic acid copy number | 1.0000 | 1.0000-1.0000 | **0.023** |
| Estimated C. pneumoniae concentration | 1.0000 | 1.0000-1.0000 | 0.246 |
| White blood cell count (×10⁹/L) | 1.1951 | 1.1162-1.2796 | **< 0.001** |
| Neutrophil percentage (%) | 1.0298 | 1.0037-1.0567 | **0.025** |
| Neutrophil count (×10⁹/L) | 1.2563 | 1.1432-1.3806 | **< 0.001** |
| Lymphocyte percentage (%) | 0.9433 | 0.9098-0.9780 | **0.002** |
| Lymphocyte count (×10⁹/L) | 0.7686 | 0.4933-1.1976 | 0.245 |
| Neutrophil-to-lymphocyte ratio (NLR) | 1.4365 | 1.2530-1.6470 | **< 0.001** |
| Eosinophil percentage (%) | 0.9226 | 0.8086-1.0526 | 0.231 |
| Eosinophil count (×10⁹/L) | 1.0755 | 0.2913-3.9702 | 0.913 |
| Hemoglobin (g/L) | 1.0038 | 0.9775-1.0308 | 0.779 |
| Platelet count (×10⁹/L) | 1.0035 | 0.9997-1.0074 | 0.074 |
| C-reactive protein (mg/L) | 1.0715 | 1.0630-1.0800 | **< 0.001** |
| Interleukin-6 (pg/mL) | 1.0656 | 1.0534-1.0779 | **< 0.001** |
| Procalcitonin (ng/mL) | 1.6516 | 0.3135-8.7029 | 0.554 |
| Serum amylase (U/L) | 1.0058 | 1.0009-1.0108 | **0.021** |
| Erythrocyte sedimentation rate (mm/h) | 1.1006 | 1.0787-1.1230 | **< 0.001** |
| Lactate dehydrogenase (U/L) | 1.0081 | 1.0047-1.0115 | **< 0.001** |
| D-dimer (ug/mL) | 1.0040 | 1.0021-1.0059 | **< 0.001** |
| Specific IgE (IU/mL) | 1.0010 | 1.0001-1.0018 | **0.028** |
| Total IgE (IU/mL) | 1.0021 | 1.0016-1.0026 | **< 0.001** |
| Right upper lobe involvement | 1.6351 | 0.8574-3.1181 | 0.135 |
| Right middle lobe involvement | 1.2535 | 0.6418-2.4483 | 0.508 |
| Right lower lobe involvement | 1.1272 | 0.6139-2.0695 | 0.699 |
| Left upper lobe involvement | 2.1312 | 0.9466-4.7980 | 0.068 |
| Left lower lobe involvement | 1.7507 | 0.8963-3.4194 | 0.101 |
| Number of lobes involved | 1.7585 | 1.3107-2.3592 | **< 0.001** |
| Bilateral lung involvement | 2.1312 | 0.9466-4.7980 | 0.068 |
| Ground-glass opacity | 2.4225 | 0.8646-6.7876 | 0.092 |
| Nodule | 1.0129 | 0.5497-1.8662 | 0.967 |
| Patchy shadow | 0.8509 | 0.4618-1.5677 | 0.605 |
| Air bronchogram | 0.4122 | 0.2072-0.8201 | **0.012** |
| Extrapulmonary symptoms | 1.0279 | 0.5344-1.9773 | 0.934 |
| Pleural effusion | 1.7333 | 0.4189-7.1720 | 0.448 |
| Allergic rhinitis | 1.9445 | 1.0431-3.6248 | **0.036** |

Note：OR: Odds Ratio; CI: Confidence Interval. Bold P-values indicate statistical significance (P < 0.05).

**Supplementary Table S5. Variables Selected by LASSO Regression with Different Lambda Criteria**

| **Variable** | **Coefficient(lambda.min)** | **Coefficient(lambda.1se)** |
| --- | --- | --- |
| Neutrophil percentage (%) | 0.0972 | 0.0612 |
| Erythrocyte sedimentation rate (mm/h) | 0.0890 | 0.0267 |
| Lactate dehydrogenase (U/L) | 0.0129 | 0.0073 |
| Duration of illness at admission (days) | 0.0413 | — |
| Hypoxemia | 0.7205 | — |
| Platelet count (×10^9^/L) | 0.0013 | — |
| Number of homogeneous sequences | -0.00002 | — |

Note: Variables are listed in order of their appearance in both lambda criteria. The lambda.min criterion selected 7 variables with higher model accuracy (97.6%), while the lambda.1se criterion selected 3 core variables with more stringent regularization (88.1% accuracy). ‘—’ indicates that the variable was not selected under the lambda.1se criterion.

**Supplementary Table S6. Performance Comparison of LASSO Models with Different Lambda Selection Criteria**

| **Model** | **Lambda Value** | **Number ofVariables** | **Accuracy (%)** |
| --- | --- | --- | --- |
| lambda.min | 0.0342 | 7 | 97.6 |
| lambda.1se | 0.0951 | 3 | 88.1 |

Note: The lambda.min criterion minimizes cross-validation error and typically selects more variables, while lambda.1se selects the most regularized model within one standard error of the minimum, resulting in a simpler model with slightly lower accuracy but better generalizability. Model performance was evaluated using 10-fold cross-validation on the training dataset.

**Supplementary Table S7. Complete Feature Ranking from SVM-RFE Analysis**

| **Rank** | **Feature Name** | **Average Rank** |
| --- | --- | --- |
| 1 | Neutrophil_Pct | 1.9 |
| 2 | ESR | 3.0 |
| 3 | Hypoxemia | 5.4 |
| 4 | LDH | 6.5 |
| 5 | Platelet | 10.2 |
| 6 | WBC | 10.5 |
| 7 | Disease_Duration | 10.8 |
| 8 | Microbial_Concentration | 10.9 |
| 9 | Uniform_Sequence | 11.9 |
| 10 | CRP | 11.9 |
| 11 | D-dimer | 12.0 |
| 12 | NLR | 13.9 |
| 13 | Total_IgE_Allergen | 14.4 |
| 14 | Total_IgE_Humoral | 14.7 |
| 15 | Eosinophil_Pct | 14.9 |
| 16 | Age | 15.0 |
| 17 | Gender | 16.9 |
| 18 | Hemoglobin | 17.6 |
| 19 | Lymphocyte_Pct | 17.7 |
| 20 | Serum_Amylase | 17.8 |
| 21 | PCT | 18.3 |
| 22 | Allergic_Rhinitis | 19.3 |
| 23 | Extrapulmonary_Symptoms | 19.6 |
| 24 | Fever | 20.2 |
| 25 | Allergy_History | 20.7 |
| 26 | Cough_Characteristics | 20.9 |
| 27 | IL-6 | 21.1 |

Note: Features are ranked based on average ranking from 10-fold cross-validation. Lower average rank indicates higher importance in the SVM-RFE model. SVM-RFE: Support Vector Machine-Recursive Feature Elimination; Average Rank: mean ranking across all cross-validation folds.

Analysis Parameters: Total features evaluated: 27; Sample size: 42 (16 without consolidation, 26 with consolidation); Cross-validation folds: 10; Kernel: Linear; Cost parameter: optimized via grid search (range: 0.1, 1, 10); Baseline accuracy: 61.9%.

**Supplementary Table S8. Complete Feature Importance Ranking from Random Forest Analysis**

| **Rank** | **Feature Name (English)** | **Importance Score** | **Category** |
| --- | --- | --- | --- |
| **1** | **LDH** | **2.608** | **High** |
| **2** | **CRP** | **2.506** | **High** |
| **3** | **ESR** | **2.179** | **High** |
| **4** | **Neutrophil_Pct** | **1.814** | **Medium** |
| **5** | **WBC** | **1.585** | **Medium** |
| **6** | **NLR** | **1.429** | **Medium** |
| 7 | Lymphocyte_Pct | 0.833 | Low |
| 8 | Uniform_Sequence | 0.734 | Low |
| 9 | Platelet | 0.681 | Low |
| 10 | IL-6 | 0.559 | Low |
| 11 | PCT | 0.550 | Low |
| 12 | Disease_Duration | 0.534 | Low |
| 13 | D-dimer | 0.439 | Low |
| 14 | Serum_Amylase | 0.396 | Low |
| 15 | Eosinophil_Pct | 0.381 | Low |
| 16 | Total_IgE_Humoral | 0.362 | Low |
| 17 | Total_IgE_Allergen | 0.328 | Low |
| 18 | Hemoglobin | 0.306 | Low |
| 19 | Age | 0.290 | Low |
| 20 | Microbial_Concentration | 0.164 | Low |
| 21 | Gender | 0.126 | Low |
| 22 | Cough_Characteristics | 0.096 | Low |
| 23 | Fever | 0.071 | Low |
| 24 | Allergic_Rhinitis | 0.069 | Low |
| 25 | Hypoxemia | 0.067 | Low |
| 26 | Allergy_History | 0.046 | Low |
| 27 | Extrapulmonary_Symptoms | 0.042 | Low |

Note: Features are ranked based on mean decrease in Gini impurity from Random Forest analysis. Higher importance scores indicate greater contribution to model prediction. Category classification: High importance (≥2.0, highlighted in light red, n=3): key predictive features; Medium importance (1.0-2.0, highlighted in light yellow, n=3): moderately important inflammatory markers; Low importance (<1.0, n=21): minor contributors.

Model Parameters: Total features evaluated: 27; Sample size: 42 (16 without consolidation, 26 with consolidation); Optimal hyperparameters: mtry=5, ntree=500; Cross-validation: 10-fold; OOB error stabilized at ~200 trees. The threshold of 2.0 for feature selection was determined based on mean Gini decrease values.

**Supplementary Table S9. Feature Importance Ranking by XGBoost Model**

| **Rank** | **Feature** | **Gain** | **Cover** | **Frequency** |
| --- | --- | --- | --- | --- |
| 1 | C-reactive protein (mg/L) | 0.3262 | 0.1541 | 0.0843 |
| 2 | Erythrocyte sedimentation rate (mm/h) | 0.2391 | 0.2345 | 0.2108 |
| 3 | Lactate dehydrogenase (U/L) | 0.1649 | 0.1765 | 0.1867 |
| 4 | Uniform sequence | 0.0854 | 0.0713 | 0.0663 |
| 5 | White blood cell count (×10⁹/L) | 0.0687 | 0.1164 | 0.1446 |
| 6 | Neutrophil percentage (%) | 0.0571 | 0.0937 | 0.1084 |
| 7 | Platelet count (×10⁹/L) | 0.0266 | 0.0543 | 0.0542 |
| 8 | Eosinophil percentage (%) | 0.0137 | 0.0251 | 0.0301 |
| 9 | Interleukin-6 (pg/mL) | 0.0108 | 0.0428 | 0.0663 |
| 10 | Disease duration (days) | 0.0069 | 0.0266 | 0.0422 |
| 11 | Serum amylase (U/L) | 0.0008 | 0.0048 | 0.0060 |

Note: Features are ranked by Gain, which measures the improvement in accuracy brought by a feature to the branches it is on. Cover measures the relative quantity of observations concerned by a feature, and Frequency indicates how often the feature is used in the trees. The top 3 features (C-reactive protein, erythrocyte sedimentation rate, and lactate dehydrogenase) cumulatively contribute 73.0% of the total Gain.

**Supplementary Table S10. Optimal Hyperparameters of XGBoost Model**

| **Parameter** | **Value** | **Description** |
| --- | --- | --- |
| nrounds | 100 | Number of boosting rounds |
| Max-depth | 3 | Maximum tree depth |
| eta | 0.1 | Learning rate (shrinkage) |
| gamma | 0 | Minimum loss reduction for split |
| colsample_bytree | 1 | Column sampling ratio per tree |
| min_child_weight | 1 | Minimum sum of instance weight in child |
| subsample | 1 | Row sampling ratio per tree |

Note: Hyperparameters were optimized using grid search with 5-fold cross-validation. An additional 10-fold stratified cross-validation was performed to evaluate the generalization performance (AUC) of the final model using the optimized hyperparameters. The three primary parameters (nrounds, max_depth, and eta) directly control model complexity and learning rate, while the remaining parameters use default values suitable for this dataset.

**Supplementary Table S11. Complete Feature Importance Ranking from LightGBM Analysis**

| **Rank** | **Feature Name (English)** | **Gain Score** | **Category** |
| --- | --- | --- | --- |
| **1** | **ESR** | **0.337** | **High** |
| **2** | **CRP** | **0.196** | **High** |
| **3** | **LDH** | **0.148** | **High** |
| **4** | **WBC** | **0.118** | **High** |
| **5** | **Neutrophil_Pct** | **0.109** | **High** |
| **6** | **Platelet** | **0.024** | **Medium** |
| **7** | **Uniform_Sequence** | **0.020** | **Medium** |
| **8** | **Eosinophil_Pct** | **0.013** | **Medium** |
| **9** | **Lymphocyte_Pct** | **0.012** | **Medium** |
| 10 | NLR | 0.009 | Low |
| 11 | Serum_Amylase | 0.004 | Low |
| 12 | D-dimer | 0.003 | Low |
| 13 | Cough_Characteristics | 0.002 | Low |
| 14 | Total_IgE_Allergen | 0.002 | Low |
| 15 | Disease_Duration | 0.001 | Low |
| 16 | IL-6 | 0.000 | Low |
| 17 | Hemoglobin | 0.000 | Low |
| 18 | Microbial_Concentration | 0.000 | Low |
| 19 | Allergic_Rhinitis | 0.000 | Low |
| 20 | Total_IgE_Humoral | 0.000 | Low |
| 21 | PCT | 0.000 | Low |
| 22 | Allergy_History | 0.000 | Low |
| 23 | Hypoxemia | 0.000 | Low |
| 24 | Fever | 0.000 | Low |
| 25 | Age | 0.000 | Low |
| 26 | Extrapulmonary_Symptoms | 0.000 | Low |
| 27 | Gender | 0.000 | Low |

**Note:** Features are ranked based on Gain values from LightGBM analysis. Gain represents the improvement in loss function contributed by each feature. Higher Gain scores indicate greater predictive importance. **Category classification:** High importance (Gain ≥ 0.1, highlighted in light red, n=5): key predictive features; Medium importance (0.01 ≤ Gain < 0.1, highlighted in light yellow, n=4): moderately important features; Low importance (Gain < 0.01, n=18): minor contributors. **Top 3 features (ESR, CRP, LDH) contributed 68.1% of cumulative gain.**

**Model Parameters:** Total features evaluated: 27; Sample size: 42 (16 without consolidation, 26 with consolidation); Optimal hyperparameters: num_leaves = 5, learning_rate = 0.01, min_data_in_leaf = 3, feature_fraction = 0.7, bagging_fraction = 0.7, lambda_l1 = 0.5, lambda_l2 = 0.5; Cross-validation: 5-fold stratified CV with grid search; Early stopping rounds: 50. Feature selection threshold: Gain>0.01 or top 15 ranked features, resulting in 15 selected features for model optimization.

# **Supplementary Table S12. Optimal Number of Clusters Evaluation**

| **Method** | **Optimal k** | **Description** |
| --- | --- | --- |
| Elbow Method | 8 | Minimizes within-cluster sum of squares |
| Silhouette Method | 2 | Maximizes average silhouette width |
| Gap Statistic | 1 | Maximizes gap statistic |
| NbClust (Majority Vote) | 2 | Consensus from 26 indices |
| Calinski-Harabasz Index | 9 | Maximizes between/within cluster variance ratio |
| **Overall Recommendation** | **2** | **Based on majority vote and clinical interpretability** |

Note: Five methods were used to evaluate the optimal number of clusters (k). The silhouette method and NbClust majority vote both recommended k = 2, which was chosen as the final clustering solution.

# **Supplementary Table S13. Clustering Quality Metrics and Parameters**

| **Metric** | **Value** |
| --- | --- |
| Number of Clusters | 2 |
| Total Sample Size | 42 |
| Clustering Variables | LDH, CRP, ESR |
| Standardization Method | Z-score normalization |
| Algorithm | K-means |
| Number of Random Starts | 100 |
| R² (Variance Explained) | 0.409 (40.9%) |

Note: LDH, lactate dehydrogenase; CRP, C-reactive protein; ESR, erythrocyte sedimentation rate. R² represents the proportion of total variance explained by the clustering solution. K-means algorithm was run with 100 random starts to ensure stability of the solution.

# **Supplementary Table S14. Summary of Sensitivity Analyses**

| **Analysis** | **Method** | **Result** | **Interpretation** |
| --- | --- | --- | --- |
| LOOCV | 42 iterations, each excluding one patient | 40/42 stable (95.2%) | Clustering solution highly robust to individual patient exclusion |
| Random seed sensitivity | 100 seeds (1–100), ≥90% overlap criterion | 100/100 identical (100%) | Solution fully reproducible regardless of initialization |
| Threshold sensitivity | Youden optimal ± 1.0, step 0.5 | Sens ≥73.1%, Spec ≥81.2% across all tested thresholds | Cut-off value is stable within clinically acceptable range |

Note: LOOCV, leave-one-out cross-validation. Seed sensitivity criterion: ≥90% of patients assigned to the same cluster as the original solution (seed = 12345).
